# Chlorine Isotope Effects from Isotope Ratio Mass Spectrometry Suggest Intramolecular C-Cl Bond Competition in Trichloroethene (TCE) Reductive Dehalogenation 

**DOI:** 10.3390/molecules19056450

**Published:** 2014-05-20

**Authors:** Stefan Cretnik, Anat Bernstein, Orfan Shouakar-Stash, Frank Löffler, Martin Elsner

**Affiliations:** 1 Institute of Groundwater Ecology, Helmholtz Zentrum München, Ingolstädter Landstr. 1, 85764 Neuherberg, Germany; E-Mails: one_fiftyone@hotmail.com (S.C.); anatbern@bgu.ac.il (A.B.); 2 Department of Earth and Environmental Sciences, University of Waterloo, Waterloo, ON N2L 3G1, Canada; E-Mail: orfan@uwaterloo.ca; 3 Department of Microbiology & Center for Environmental Biotechnology, University of Tennessee, Knoxville, TN 37996-2000, USA; E-Mail: frank.loeffler@utk.edu; 4 Department of Civil and Environmental Engineering, University of Tennessee, Knoxville, TN 37996-2000, USA; 5 Oak Ridge National Laboratory (UT-ORNL) Joint Institute for Biological Sciences (JIBS) and Bioscience Division, Oak Ridge National Laboratory, Oak Ridge, TN 37831, USA

**Keywords:** reductive dehalogenation, chlorinated ethenes, trichloroethene, biodegradation, organohalide respiration, dechlorination mechanism, regioselectivity, vitamin B_12_, reductive dehalogenase

## Abstract

Chlorinated ethenes are prevalent groundwater contaminants. To better constrain (bio)chemical reaction mechanisms of reductive dechlorination, the position-specificity of reductive trichloroethene (TCE) dehalogenation was investigated. Selective biotransformation reactions (i) of tetrachloroethene (PCE) to TCE in cultures of *Desulfitobacterium* sp. strain Viet1; and (ii) of TCE to *cis*-1,2-dichloroethene (*cis*-DCE) in cultures of *Geobacter lovleyi* strain SZ were investigated. Compound-average carbon isotope effects were −19.0‰ ± 0.9‰ (PCE) and −12.2‰ ± 1.0‰ (TCE) (95% confidence intervals). Using instrumental advances in chlorine isotope analysis by continuous flow isotope ratio mass spectrometry, compound-average chorine isotope effects were measured for PCE (−5.0‰ ± 0.1‰) and TCE (−3.6‰ ± 0.2‰). In addition, position-specific kinetic chlorine isotope effects were determined from fits of reactant and product isotope ratios. In PCE biodegradation, primary chlorine isotope effects were substantially larger (by −16.3‰ ± 1.4‰ (standard error)) than secondary. In TCE biodegradation, in contrast, the product *cis*-DCE reflected an average isotope effect of −2.4‰ ± 0.3‰ and the product chloride an isotope effect of −6.5‰ ± 2.5‰, in the original positions of TCE from which the products were formed (95% confidence intervals). A greater difference would be expected for a position-specific reaction (chloride would exclusively reflect a primary isotope effect). These results therefore suggest that both vicinal chlorine substituents of TCE were reactive (intramolecular competition). This finding puts new constraints on mechanistic scenarios and favours either nucleophilic addition by Co(I) or single electron transfer as reductive dehalogenation mechanisms.

## 1. Introduction

Chlorinated organic compounds have natural and anthropogenic sources and are represented in nearly every organic chemical class [1]. Much fundamental interest is directed at the underlying reaction chemistry of the C-Cl bond [2,3,4]. The widespread industrial application of chlorinated hydrocarbons as solvents, chemical intermediates and pesticides resulted in environmental contamination, with adverse effects on drinking water quality and ecosystem and human health [5,6,7]. A specific focus has therefore been on their reductive dehalogenation to non-halogenated hydrocarbons, where detoxification is achieved by reductive cleavage of the carbon-chlorine bonds. Evolution has brought forward specialized microorganisms that perform organohalide respiration by using chlorinated hydrocarbons for energy conservation [8].

**Scheme 1:**
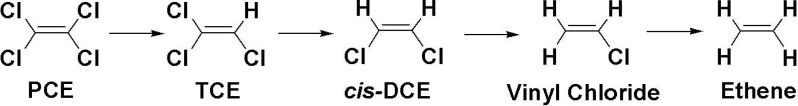
Typical stepwise reaction sequence in microbial dechlorination of PCE leading to less chlorinated ethenes and to non-toxic ethene.

Considering the importance of biotic reactions involved in the degradation of chlorinated hydrocarbons, surprisingly little is known about the underlying reaction mechanisms. Even the detailed reductive dehalogenation mechanisms of tetrachloroethene (PCE) and trichoroethene (TCE)—the two most abundant dry cleaning and degreasing agents and notorious groundwater pollutants—remain imperfectly understood. Some bacteria produce *trans*-DCE or 1,1-DCE in this reaction [9], but the typical case is the selective formation of *cis*-DCE as the bottleneck of microbial dehalogenation, which is a major problem in remediation strategies (Scheme 1) [10].

**Scheme 2:**
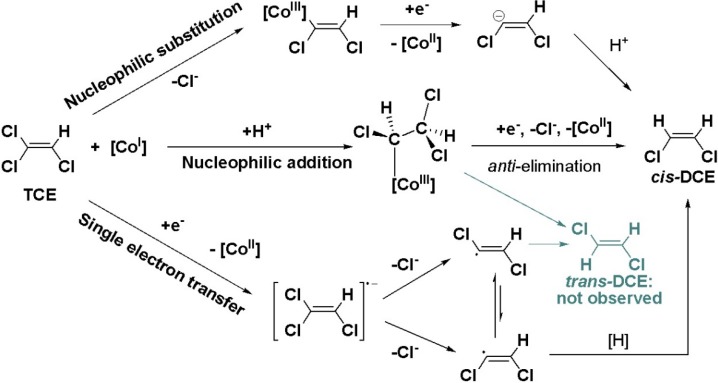
Proposed degradation pathways of TCE catalyzed by cobalamin.

On the most fundamental level, this product formation depends on the initial reaction step of cob(I)alamin (coenzyme B_12_), the transition-metal cofactor present in reductive dehalogenases [11]. It is unclear whether the underlying dehalogenation reaction involves nucleophilic addition by Co(I), nucleophilic substitution by Co(I) or a radical mechanism involving a single electron transfer (SET) step from Co(I) to the organohalide (Scheme 2) [12,13,14,15,16,17,18].

Specifically, it remains unclear whether the transformation of TCE to *cis*-DCE is stereoselective at the geminal chlorine substituent in *E*-position—as one would presume for a nucleophilic substitution mechanism [19]—or whether both geminal *E*- and *Z*-positions are involved—as brought forward for SET by computational results from Nonnenberg *et al.* resulting in the formation of radical intermediates [20]. Involvement of both germinal chlorine substituents would also be expected for the pathway of nucleophilic addition, as suggested by a computational study by Pratt and van der Donk [21]. In the light of the selective formation of *cis*-DCE, a direct, complementary line of evidence is therefore warranted, which indicates whether one or two carbon-chlorine bonds are reactive in TCE.

A potential solution are measurements of kinetic isotope effects (KIEs), either on reacting bonds (primary isotope effects) or on adjacent bonds (secondary isotope effects). If only one C-Cl bond in TCE is reactive, a primary isotope effect would occur specifically in this position leading to a pronounced difference to the other positions where only secondary isotope effects would be expected. In contrast, if two positions are reactive, they would take turns in the reaction so that both would reflect a combination of primary and secondary effects. If isotope effects in both positions are compared, a smaller difference would therefore be expected. On the most fundamental level, however, general knowledge about typical primary and secondary isotope effects of chlorine would first be warranted, since so far only very limited knowledge exists on isotope effects in reductive dechlorination of chlorinated ethenes. 

To close this research gap, KIEs on specific positions in a target molecule must be determined. Typical techniques are isotope labelling, or determination of position-specific KIEs by NMR-measurements [22]. However, these approaches do not work well for measurement of chlorine isotope effects in chlorinated ethenes. Even though the stable isotopes of chlorine (^35^Cl and ^37^Cl) are NMR active, they show broad chemical shifts and poor precision in signal integrations so that position-specific ^37^Cl/^35^Cl isotope analysis with NMR is challenging. [23] In addition, the method is not suitable to investigate biodegradation because samples contain diluted compound mixtures and NMR analysis requires large amounts of pure target compounds. Finally, position-specific isotopic labeling is impossible for PCE, where all chlorine substituents are chemically equivalent [24].

A potential solution to the problem is offered by recent instrumental developments in gas chromatography-isotope ratio mass spectrometry (GC-IRMS). Compounds are separated by gas chromatography and isotopologue ion multiplets of individual chlorinated ethene molecules are recorded simultaneously in dedicated Faraday cups of isotope ratio mass spectrometers [25,26]. Measurements of changes in chlorine isotope ratios have therefore become accessible even in complicated reaction mixtures observed in environmental systems. However, the measured isotope effects from such GC-IRMS methods typically reflect the compound average of the target compound. 

Even though the recent introduction of chlorine isotope GC-IRMS—combined with routine carbon isotope GC-IRMS—has brought about first dual (carbon and chlorine) isotope effect investigations, these studies have, therefore, only targeted *compound-average* isotope effects of the *reactant* [27,28,29,30]. While they could delineate similarities and differences between experimental systems (different microorganisms, chemical reactants) [29] direct insight into underlying mechanisms remained elusive. 

In this study, we take advantage of the fact that Cl^−^ is released during reductive dechlorination. Since the chlorine substituents of the parent chlorinated ethene end up in different products (*i.e.*, Cl^−^ and the less chlorinated ethene), they are subject to different isotope effects (primary effects in the cleaved bond, secondary effects in non-reacting positions). Information on the magnitude of either *position-specific* isotope effect may therefore be retrieved from analysis of isotope ratios in the *products* (Cl^−^ and the less chlorinated ethene). This approach was discussed in previous work [31] and was pursued in a recent experimental study by Kuder *et al.* [30]. There, it was *a priori* excluded that more than one C-Cl bond is reactive in TCE—in contradiction to the scenarios of Scheme 1. Since this directly affected all further conclusions of this previous study—including estimates of secondary chlorine isotope effects [30]—resultant mechanistic conclusions were, unfortunately, biased and reliable insight based on Cl-isotope effect interpretations was not possible.

In this study, a more rigorous evaluation was enabled by (i) investigations of one-step dehalogenation reactions only and (ii) application of an appropriate mathematical framework. Cultures of *Desulfitobacterium* sp. strain Viet1, a PCE-to-TCE dechlorinator [32], were used to determine the typical magnitude of primary and secondary chlorine isotope effects. Further, cultures of *Geobacter lovleyi* strain SZ, a PCE/TCE-to-cis-DCE dechlorinator [33], were used to specifically investigate whether one or two C-Cl bonds are reactive in TCE as non-symmetric molecule. Mathematical equations were derived for reactant and product isotope ratios to model chlorine isotope trends and to extract primary and secondary chlorine isotope effects. This approach provided a first benchmark how chlorine isotope data can be interpreted in typical scenarios of reductive dechlorination of chlorinated ethenes. Another aim was to explore if information on one *versus* two reactive positions may be obtained and could be useful to constrain the number of possible reaction mechanisms for reductive TCE dehalogenation.

## 2. Experimental Section

### Biodegradation Experiments and Carbon and Chlorine Isotope Analysis

Selective reductive dechlorination of PCE to TCE was accomplished in anaerobic biodegradation of PCE with the Firmicute *Desulfitobacterium* sp. strain Viet1, and selective transformation of TCE to *cis*-DCE was facilitated by the Deltaproteobacterium *Geobacter lovleyi* strain SZ. The cultures were grown following established procedures inside an anoxic glove box in glass bottles (250 mL) equipped with Mininert valves (Supelco, Bellefonte, PA, USA). The bottles were amended with 10 µL of neat PCE. After four days of shaking, inoculation was carried out by adding 20 mL of the active culture, which was previously grown in a similar medium. Liquid samples of 7 mL were collected at given time points within 48 h after inoculation until the initial amount of PCE or TCE (45 mg/L and 90 mg/L, respectively) had been dechlorinated. The samples were taken for (i) compound-specific isotope analysis (CSIA) of carbon and chlorine in chlorinated ethenes by GC-IRMS and (ii) concentration analysis using a gas chromatograph equipped with flame ionization detector (GC-FID). Limit of detection in these concentration measurements for the chlorinated ethenes were below 0.05 µg/L corresponding to less than 0.1% of the initial concentrations. This includes 1,1-DCE and *trans*-DCE as potential dechlorination products, which were not observed in any of the reactions. Abiotic controls were treated with an identical procedure but without inoculation of an active microbial culture. Concentrations in these controls did not decrease in significant amounts. The analytical uncertainty 2σ was ±0.5‰ for carbon isotope analysis and ±0.2‰ for chlorine isotope analysis. Detailed descriptions of experimental and analytical methods are provided in the Supporting Information (S.I.).

## 3. Mathematical Equations for Fitting Substrate and Product Isotope Ratios (I): Compound-Average Isotope Effects

Mass spectrometry can measure the proportion of different stable isotopes of element E in a given molecule. When looking at a given molecular position, this ratio of heavy isotopes ^h^E to light isotopes ^l^E, denoted with R_0_ for the starting material, typically changes during the progress of a reaction to a different ratio R_t_ at time t:

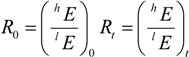



Owing to the kinetic isotope effect (KIE), slower reacting isotopes become enriched during the reaction compared to the original starting material. This KIE_E_ is given by the ratio of rate constants of the light isotope ^l^k and heavy isotope ^h^k:

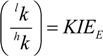
(1)


For the sake of simplicity, we stick to this this definition even though, strictly speaking, bacterial transformation gives isotope effects on (V/K)—V: maximum enzyme velocity, K: Michaelis-Menten constant—rather than on elementary rate constants k [34].

### 3.1. Compound-Average Isotope Effects from Reactant Values

The proportion of *R_t_/R_0_* is well established to depend on the remaining fraction *f* of the starting material and the KIE according to [34]:

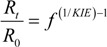
(2)


In the case of compound-specific isotope analysis, *R* represents the isotope ratio as a compound average *R_Compound-Average_*. This means that also the kinetic isotope effect is obtained as a compound average *KIE_Compound-Average_* [35]:

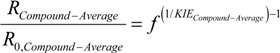
(3)


The exponent of Equation (3) can alternatively be called enrichment factor ε and 1/KIE be named fractionation factor α with the relationship [36]:

(1 / *KIE_Compound-Average_*) − 1 = α − 1 = ε (4)

A *KIE_Compound-Average_* of 1.005, for example, corresponds to ε = −0.005 = −5‰, expressing a situation, in which molecules with an additional heavy isotope react on average by 5‰ slower than the respective lighter isotopologue. Combination of Equations (4) and (3) results in:

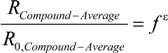
(5)


The isotope ratios of the heavy (^h^E) and light (^l^E) element in a compound are typically stated relative to reference materials, (Vienna Pee Dee Belemnite (VPDB) for carbon, Standard Mean Ocean Chloride (SMOC) for chlorine) and the isotope ratio of a substance is typically expressed in the delta notation:

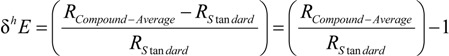
(6)


Equation (6) can be rearranged to:

*R_Compound-Average_* = (δ*^h^E* + 1) · *R_Standard_* (7)

This can be introduced into Equation (5) according to:

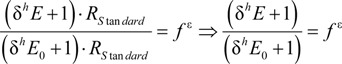
(8)


Equation (8) is typically used in its logarithmic form as the common Rayleigh equation:

ln (δ*^h^E* + 1) - ln (δ*^h^E*_0_ + 1) ≈ δ*^h^E* − δ*^h^E*_0_ = ε ln *f* ⇒ δ*^h^E* = δ*^h^E*_0_ + ε ln *f* (9)

The obtained epsilon (ε) represents the isotope fractionation as a compound average and therefore expresses the average positions *p* of the element in the target compound, including primary as well as secondary positions:

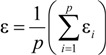
(10)


### 3.2. Expressions for Product Isotope Values

For the products formed during the reaction, an isotopic mass balance must be fulfilled in a closed system:

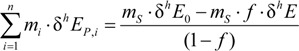
(11)


Here, the reactant contains *m_S_* atoms of element *E* in its structure. *δ^h^E_0_* is the original reactant isotope ratio, whereas *δ^h^E* is the ratio when reaction has occurred so that only a fraction *f* of reactant remains. A fraction of (1 − *f*) has then been converted to one or more (up to *n*) products; *m_i_* is the number of atoms of E inside the structure of product *i*, *δ^h^E_P,i_* is the respective product’s isotope value. 

### 3.3. Carbon Isotope Effects from Product Values

In the conversion from PCE to TCE or from TCE to *cis*-DCE, the two carbon atoms present in the reactant are passed on to the product. Therefore, in the case of carbon, Equation (11) simplifies to:

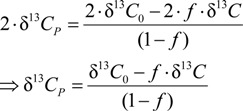
(12)


An equation that allows fitting product isotope trends is obtained by combination of Equation (12) with Equation (9) to yield

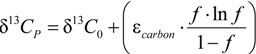
(13)


The parameter ε*_carbon_* from product data is identical to ε in Equation (9) for substrate data, because all carbon isotopes are transferred from reactant to product so that the product isotope curve reflects the enrichment trend of the original atoms in the reactant.

### 3.4. Chlorine Isotope Effects from Product Values

When chlorine isotope values are measured, reductive dechlorination of chlorinated ethenes releases chloride and simultaneously forms the less chlorinated ethene so that two chlorine-containing products are formed at the same time (*n* = 2). This circumstance generates information about primary and secondary chlorine isotope effects, as derived in the following section.

## 4. Mathematical Equations for Fitting Product Data (II): Insight into Primary and Secondary Chlorine Isotope Effects

### 4.1. Case 1—PCE: Indistinguishable Molecular Positions

#### 4.1.1. General Equations

In the case of PCE, the molecular positions of all atoms are chemically equivalent so that the same chlorine atoms may potentially end up in TCE or as Cl^−^. Accordingly, isotopes partition according to the kinetic isotope effects associated with the formation of either product *P_i_* with α*_i_ = 1/KIE_i_*. As a consequence, their isotope ratios relate according to:

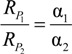
(14)


With Equation (7), the fractionation factors α*_i_* can be expressed in the delta notation as:

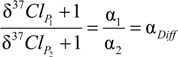
(15)
with α*_Diff_* expressing the ratio between the primary isotope effect (in the formation of Cl^−^) and the average secondary isotope effects (in the three molecular positions that become TCE). This equation can be rearranged and simplified according to:

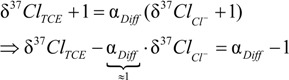
(16)

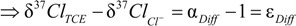
(17)


Accordingly, the difference between primary and secondary isotope effects ε*_Diff_* is directly obtained from product isotope values, because chlorine isotope ratios of Cl^−^ and TCE are always separated by ε*_Diff_*.

For the reaction of PCE with four chlorine atoms to TCE with three chlorine atoms and Cl^−^ the enrichment trends of these products must follow Equation (17), as well as an isotopic mass balance. According to the derivation in the supporting information, the following equations apply:

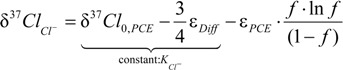
(18)

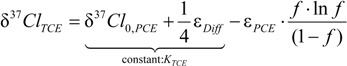
(19)


When *f* approaches 1 in this expression (*i.e.*, in the beginning of the reaction), the last term approaches +ε*_PCE_* in Equations (18) and (19). As a consequence, the intercept (A) between PCE and the TCE formed, and the intercept (B) between PCE and the chloride released are determined by:

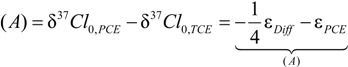
(20)

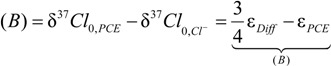
(21)


For interpretation of the experimental PCE degradation data, the product isotope trends of chloride and TCE were fitted in Sigma Plot according to Equations (18) and (19), respectively, with ε*_PCE_* and ε*_Diff_* as fitting parameters.

#### 4.1.2. Interpretation of Product Isotope Enrichment Trends (ε_Chlorine_) and intercepts (ε_Difference_) for PCE

In the case of PCE, ε*_Diff_*—the intercept of the product curves—provides information about primary and secondary isotope effects. The parameter ε_PCE_, in contrast, relates to changes in the four chemically equivalent positions of PCE (average of primary and secondary isotope effects) and is the same in fits of δ^37^*Cl_PCE_* [Equation (9)], δ^37^*Cl_Cl−_* [Equation (20)] and δ^37^*Cl_TCE_* [Equation (21)], for the same reasons as discussed above for carbon.

### 4.2. Case 2—TCE: Distinguishable Molecular Positions

**Scheme 3:**
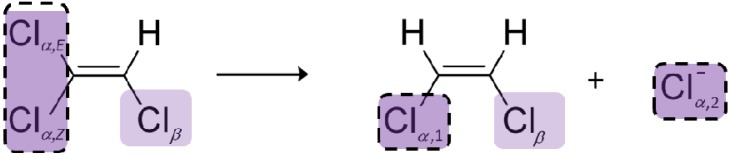
One step reductive dechlorination of TCE with possible locations of primary isotope effects (dotted line) and the location of a secondary isotope effect (Cl_β_).

In contrast to PCE, molecular positions in TCE are not chemically equivalent. In addition, formation of *cis*-DCE is clearly regioselective. No mechanism is known which would convert 1,1-dichloroethene into 1,2-dichloroethene intermediates so that *cis*-DCE can only be formed by cleavage of a C-Cl bond in the geminal α-positions and not in the β-position (see Scheme 3). Otherwise 1,1-dichloroethene would be detected which is not the case for *Geobacter lovleyi* strain SZ [33]. A primary isotope effect is therefore expected in the reacting bond in α-position and a secondary isotope effect in the other germinal bond in α-position that is not cleaved. A secondary isotope effect is also expected in the vicinal bond at Cl_β_ because it does not experience C-Cl bond cleavage.

Although the two chlorine atoms in *cis*-DCE are chemically equivalent, they have a different history (see Scheme 3). One derives from the β-position of TCE—in which ^37^Cl/^35^Cl ratios change due to a secondary isotope effect—and one from the geminal α-positions of TCE, in which ^37^Cl/^35^Cl ratios may change due to a combination of primary and secondary effects.

Since no *trans*-DCE is observed, it seems an intuitive interpretation that Cl_α,*E*_ should be the only reactive position, in analogy to the considerations for Cl_β_ above. Previous studies, however, considered that selective formation of *cis*-DCE may be caused by cleavage of either C-Cl bond in the α‑positions followed by selective interconversion of *cis*-/*trans*-dichloroethene intermediates, as illustrated in Scheme 2 [20,37]. If two reactive positions are considered, one can therefore define

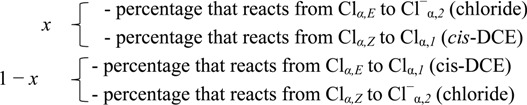

With *x* = 1 or *x* = 0, the reaction would follow a position-specific cleavage; however with 1 > *x* > 0 two positions would be involved. According to the derivation shown in the Supporting Information, the enrichment trends in chlorine isotope values of *cis*-DCE and chloride can be expected to be described by *individual* enrichment factors according to:

*ε_TCE_*_−> chloride_ = (*x·ε_α,E_* + (1 − *x*)·*ε_α,Z_*) (22)

for the enrichment trend in chloride isotope data, and

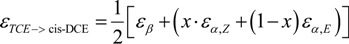
(23)
for the enrichment trend in *cis*-DCE isotope data. Here, ε_α,*E*_, ε_α,*Z*_, and ε_β_ are the position-specific isotope effects in the different bonds according to Scheme 3 (for interpretations see below). The situation is therefore different from the PCE case where both products (TCE and chloride) were formed from indistinguishable molecular positions of PCE so that their isotope enrichment trends reflected, and could be fitted by, the *same* enrichment factor ε*_PCE_*. In contrast, distinguishable chlorine atoms are present in TCE so that ε*_TCE->chloride_* and ε*_TCE->cis-DCE_* are different. They can be obtained by fitting experimental chloride isotope data according to:

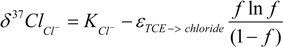
(24)
And:

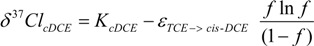
(25)


The introduced constants *K* in Equations (24) and (25) are fitting constants in Sigma Plot for the respective intercept of each product. For their mathematical definition and interpretation see below, as well as the derivatization in the Supporting Information.

#### 4.2.1. Interpretation of the Product Curve Enrichment Trends ε_TCE->chloride_ and ε_TCE->cis-DCE_ for TCE

The parameters ε*_TCE->chloride_* and ε*_TCE->cis-DCE_* describe the enrichment trend (*i.e.*, the steepness) of δ^37^Cl curves of chloride and *cis*-DCE. Intriguingly, these parameters allow a glimpse on ε_α,*E*_, ε_α,*Z*_ and ε_β_ the position-specific isotope effects, which can tell whether only one, or both positions may react. Specifically, ε_α,*E*_ is a weighted average of ε_α,*E primary*_ and ε_α,*E secondary*_. Here, ε_α,*E primary*_ is defined as the primary isotope effect when position E reacts to release chloride (percentage *x*), and ε_α,*E secondary*_ is the secondary isotope effect in E when position Z reacts to release chloride (percentage (1 − *x*)):

*ε_α,E_* = *x·ε_α,E,prim_* + (1 − *x*)·*ε_α,E,sec_* = *ε_α,E,sec_* + *x·ε_Diff,α,E_* (26)

In the same way ε_α__,Z_ is expressed as

*ε_α,Z_* = (1 − *x*)·*ε_α,Z,prim_* + *x·ε_α,Z,sec_* = *ε_α,Z,sec_* + (1 − *x*)·*ε_Diff,α,Z_* (27)

Substitution into Equation (26) gives:

*ε_TCE_*_−> chloride_ = *x·ε_α,E_* + (1 − *x*)·*ε_α,Z_* = *x* [*x·ε_α,E,prim_* + (1 − *x*)·*ε_α,E,sec_*] + (1 − *x*) [(1 − *x*)·*ε_α,Z,prim_* + *x·ε_α,Z,sec_*] (28)

In the same way, the contribution of *x·*ε_α__,*Z*_ + (1 − *x*)ε_α__,*E*_ in Equation (26)—the one which describes the chlorine atoms of *cis*-DCE stemming from the α-positions of TCE—is given by

*x·ε_α,Z_* + (1 − *x*)·*ε_α,E_* = *x* [(1 − *x*)·*ε_α,Z,prim_* + *x·ε_α,Z,sec_*] + (1 − *x*) [*x·ε_α,E,prim_* + (1 − *x*)·*ε_α,E,sec_*] (29)

**Figure 1:**
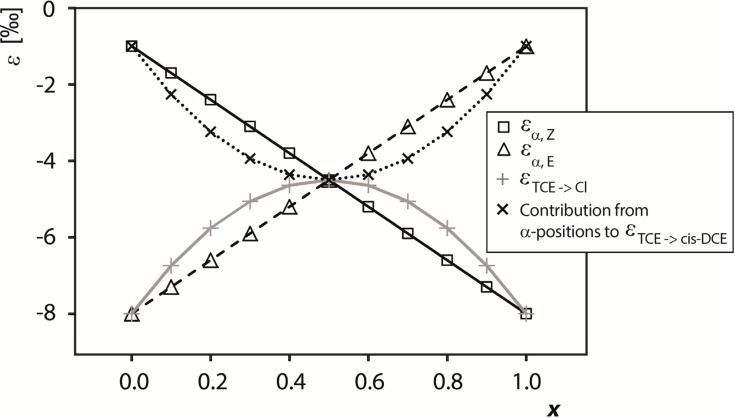
Representation of parameters ε_α,*E*_, ε_α,*Z*_, ε*_TCE->chloride_* and ε*_TCE->cis-DCE_* in dependence of *x*, the percentage of Cl^−^_α,*E*_ reacting to Cl^−^. Assumed values for primary and secondary isotope effects were ε*_primary_* = −8‰ and ε*_secondary_* = −1‰ in both positions. Similar scenarios are obtained if the values are allowed to vary between the positions (see S.I.).

Figure 1 visualizes how both contributions depend on *x* (*i.e.*, the percentage of Cl_α,*E*_ reacting to Cl) assuming exemplary numeric values for chlorine isotope effects (ε*_primary_* = −8‰ (KIE_Cl_ = 1.008) and ε*_secondary_* = −1‰ (KIE_Cl_ = 1.001)), which are further assumed to be identical in both positions (*Z* and *E*). For qualitatively similar trends with other numerical scenarios see the supporting information.

#### 4.2.2. Contributions from α-Positions to ε*_TCE->chloride_* and ε*_TCE->cis-DCE_*

Figure 1 illustrates that ε_α,*E*_ and ε_α,*Z*_ vary linearly with *x*. In contrast, a non-linear variation with *x* occurs for ε*_TCE->chloride_*, as well as for the α-position contribution of ε*_TCE‑>cis-DCE_* for the following reasons. If more chloride is formed from one position (i) ε_α_ is stronger in the position from which more chloride is formed with a primary isotope effect; (ii) in addition, more atoms in these positions are released as chloride so that the product curve of chloride more strongly reflects this higher enrichment. The opposite trend can be observed in the product curve of *cis*-DCE. The combination of both contributions (i) and (ii) therefore lends the curves of ε*_TCE‑>chloride_* and ε*_TCE->cis-DCE_* their non-linear shape. In the case of *x* = 0.5, finally, both contributions are identical because the isotope enrichment trend from either position is passed on in equal parts to both products (true intramolecular competition; this corresponds to the example of PCE, where TCE and chloride also reflect the average isotope enrichment trend of the reactant.

Figure 1, therefore, predicts that ε*_TCE->chloride_* and the contribution of *x*·ε_α,*Z*_ + (1 − *x*)ε_α,*E*_ to ε*_TCE‑>cis‑DCE_* are very sensitive indicators whether the reaction of TCE involves two or only one reactive C-Cl bond(s). As illustrated in Figure 1, if both bonds are involved, the difference between ε*_TCE->chloride_* and ε*_TCE->cis-DCE_* is smaller than predicted for a typical difference between primary and secondary isotope effects. In contrast, only in the case that one position reacts (*i.e.*, when *x* approaches 0 or 1) the positions in TCE can be strictly separated into one reactive chlorine substituent (experiencing a primary isotope effect) and two non-reactive chlorine substituents (experiencing secondary isotope effects). It is therefore only in this case that the enrichment trend of the two product curves reflects a typical difference between primary and secondary isotope effects, because only in this case is ε*_TCE->chloride_* = ε*_primary_* and ε*_TCE‑>cis‑DCE_* = ε*_secondary_*.

#### 4.2.3. Interpretation of Intercepts K for the TCE Case

As illustrated in the supporting information, the difference K in chlorine isotope signatures of the initially formed chloride and *cis*-DCE at *f* = 1 is influenced by two factors. (i) The initial chlorine isotope ratio(s) δ^37^Cl_0,i_ of the position(s), from which the respective product is formed (in contrast to PCE, the positions in TCE are not chemically equivalent and may show relevant variations of ^37^Cl/^35^Cl between each other); (ii) the kinetic isotope effect from the reaction (primary for Cl^−^, secondary for *cis*-DCE). 

Even though this kinetic isotope effect information is desirable, a direct interpretation like in the case of PCE is not possible because TCE internal isotope distributions cannot experimentally be determined. In contrast to interpretations of the PCE data, insight into position-specific chlorine isotope effects of TCE is therefore *not* given by the intercepts of Equations (24) and (25), but by the parameters ε*_TCE->chloride_* and ε*_TCE‑>cis‑DCE_*. Specifically, while in the case of PCE the parameter ε*_PCE_* is the same for all product species, ε*_TCE->chloride_* and ε*_TCE‑>cis-DCE_* reflect isotope effects in the positions of TCE, from which the respective products are formed. These product enrichment trends are, therefore, a sensitive indicator whether the reaction of TCE involves one or two reactive positions (see Figure 1).

## 5. Results and Discussion

### 5.1. Compound-Specific Carbon Isotope Effects in Reductive Dehalogenation of PCE to TCE by Desulfitobacterium sp. Strain Viet1 and of TCE to cis-DCE by Geobacter Lovleyi Strain SZ

**Figure 2:**
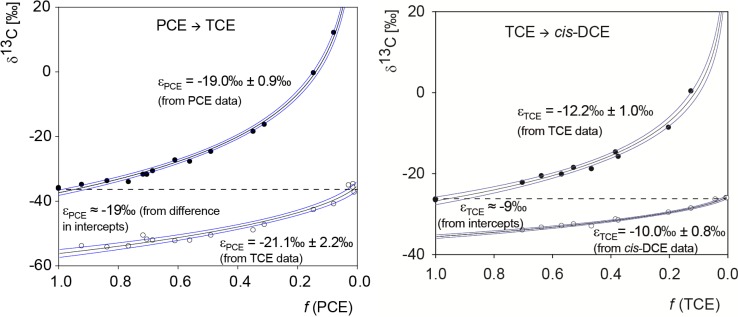
Carbon isotope data from degradation of PCE to TCE by *Desulfitobacterium* sp. strain Viet1, and of TCE to *cis*-DCE by *Geobacter lovleyi* strain SZ.

Selective reductive dechlorination of PCE to TCE and chloride was performed with the microorganism *Desulfitobacterium* sp. strain Viet1, whereas *Geobacter lovleyi* strain SZ converted TCE to stoichiometric amounts of *cis*-DCE and inorganic chloride (note that the TCE data of the latter experiment are identical to those reported in Cretnik *et al.* [29]). Evaluation of compound-specific carbon isotope fractionation during reductive dechlorination of chlorinated ethenes gave fairly consistent results by application of the Rayleigh equation in its different forms. The carbon isotopic enrichment factor ε of the transformation was either directly obtained from the difference for the very first product fraction at *f* = 1 (from intercepts: ε*_PCE_* ≈ −19‰, ε*_TCE_* ≈ −9‰ see Figure 2). Alternatively, since the product contained less ^13^C than the substrate from which it was formed, ^13^C/^12^C increased in the remaining reactant pool. Evaluation of reactant isotope data according to the Rayleigh equation [Equation (9)] was therefore an alternative way of determining ε (from reactant data: ε*_PCE_* = −19.0‰ ± 0.9‰, ε*_TCE_* = −12.2‰ ± 1.2‰). Finally, since this enrichment trend was reflected also in the steepness of the product δ^13^C curves, ε could alternatively be determined from product isotope data according to Equation (13): ε*_PCE_* = −21.1‰ ± 2.2‰ (from TCE data); ε*_TCE_* = −10.0‰ ± 0.8‰ (from *cis*-DCE data, see Figure 2). 

After complete conversion at *f* = 0, the experimental data confirm that the product isotope signature has the same carbon isotope ratio as the starting material when the isotopic mass balance was closed.

### 5.2. Compound-Specific and Position-Specific Chlorine Isotope Effects in Reductive Dehalogenation of PCE to TCE by Desulfitobacterium sp. Strain Viet1

For the selective reductive dechlorination of PCE to TCE and chloride by *Desulfitobacterium* sp. strain Viet1 chlorine isotope signatures were measured in PCE and TCE, whereas chloride isotope signatures were calculated based on the isotopic mass balance (see S.I.).

**Figure 3:**
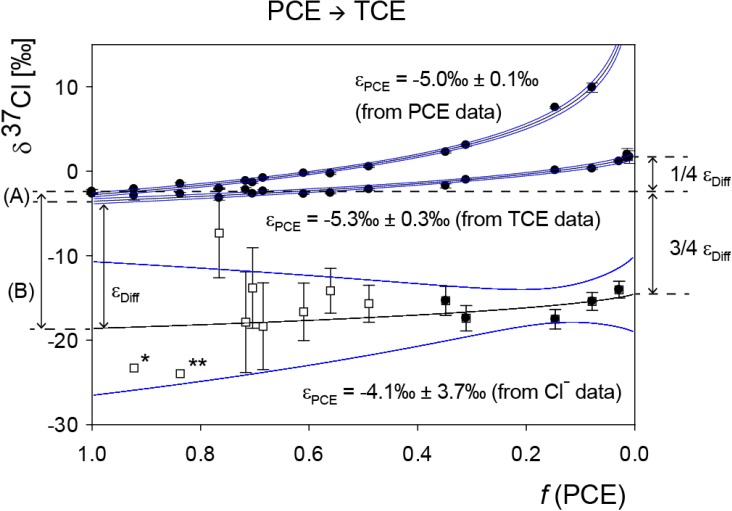
Chlorine isotope data of PCE degradation to TCE and chloride by *Desulfitobacterium* sp. strain Viet1 including fits in Sigma Plot according to the presented mathematical approach and 95% confidence intervals of the nonlinear regressions (for regression data see S.I.). Uncertainties for δ^37^Cl_Cl_^−^ were calculated by error propagation including uncertainties in δ^37^Cl_PCE_, δ^37^Cl_TCE_ and *f*(PCE) (see S.I.). Resultant uncertainties of the first two data points (error bars not shown) were 108‰ (*) and 24‰ (**). For regressions only data points with standard errors smaller than 2‰ were considered (black symbols).

Enrichment factors ε_PCE_ were obtained from PCE reactant data (−5.0‰ ± 0.1‰) according to Equation (9), from chloride product data (−4.1‰ ± 3.7‰) according to equation (18), and from TCE product data (−5.3‰ ± 0.3‰) according to Equation (19) (uncertainties are 95% confidence intervals). This confirms that the products TCE and chloride reflect the chlorine isotope enrichment trend of the four chemically equivalent positions of PCE from which they are formed. After full conversion at *f* = 0, the isotope signature of the released chloride must show an offset of −3/4ε*_Diff_* compared to the initial isotope signature of PCE, and the isotope signature of the formed TCE must show an offset of 1/4ε*_Diff_*, which the data confirm (Figure 3).

**Figure 4:**
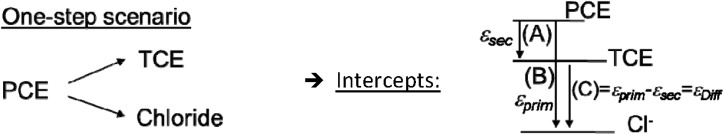
Interpretation of chlorine isotope data in a one-step scenario with respect to intercepts between PCE, TCE and chloride of the applied mathematical fits.

In contrast to the isotope pattern of carbon, these intercepts between PCE as starting material and the instantaneously formed products reveal additional information. In the case of a one-step scenario, the secondary and primary isotope effects are accessible from the intercepts according to Figure 4. The secondary chlorine isotope effect with ε*_sec_* of −1.0‰ ± 0.5‰ (standard error) is obtained in the intercept (A) as average of the three non-reacted chlorine substituents that remain in TCE. The primary chlorine isotope effect of −16.0‰ ± 4.9‰ (standard error) is extracted from the isotope signature of the instantaneously formed chloride at the beginning of the reaction in the intercept (B). 

In an alternative approach the primary isotope effect may be extracted in higher precision when considering that the enrichment factor of PCE is a weighted average of primary and secondary effects:

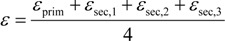
(30)


The average secondary isotope effect is further given by

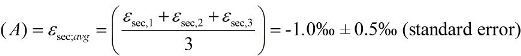
(31)
so that the primary isotope effect in PCE can be calculated:

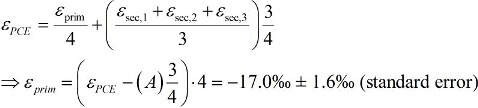
(32)


**Figure 5:**
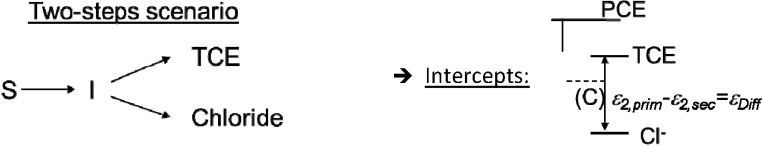
Interpretation of chlorine isotope data in a two-step scenario with respect to intercepts between PCE, TCE and chloride of the applied mathematical fits.

In contrast, if a two-step scenario prevails, no *absolute* values are obtained, but instead only the *difference* between primary and secondary isotope effects is accessible from the intercepts of the two products TCE and chloride as illustrated in Figure 5.

In this case, the observed isotope effect of PCE with −5.0‰ ± 0.1‰ reflects the first step to the intermediate (I). A second rate-limiting step forms TCE and Cl^−^. The difference between primary and secondary chlorine isotope effects for the second step is obtained from the difference of the intercepts (ε*_Diff_*) with −16.3‰ ± 1.4‰ (standard error). 

Independent of the prevailing scenario, the difference between primary and secondary isotope effects in reductive dehalogenation of PCE by *Desulfitobacterium* sp. strain Viet1 can therefore be determined as −16.3‰ ± 1.4‰. This indicates that primary chlorine isotope effects are of significantly larger magnitude than secondary isotope effects. In the case of the first scenario (one-step reaction), the exact value of the primary isotope effect (−17.0‰ ± 1.6‰) would even be directly accessible, as well as the average of all secondary isotope effects (−1.0‰ ± 0.5‰), which would be smaller by an order of magnitude. 

### 5.3. Compound-Specific and Position-Specific Chlorine Isotope Effects in Reductive Dehalogenation of TCE to cis-DCE by Geobacter lovleyi Strain SZ

*Geobacter lovleyi* strain SZ converted TCE to stoichiometric amounts of *cis*-DCE and inorganic chloride. Compound-average chlorine isotope values were measured for TCE and *cis*-DCE, and calculated for chloride based on the closed isotopic mass balance (see S.I.).

In the case of TCE, the intercepts do not provide useful information about primary and secondary isotope effects because the three positions are distinguishable and TCE may have an unequal isotope distribution, which cannot be directly measured. For instance, if the reactive position in TCE contains more ^37^Cl/^35^Cl than the average molecule, a respective “lighter” signature would be found in the formed *cis*-DCE because the produced chloride pool would contain more ^37^Cl/^35^Cl. Such a scenario would bias the interpretation of intercepts. The following discussion is therefore only based on the fitted parameters of ε, which are independent of the intercepts in the isotope patterns. Since these parameters reflect enrichment trends in molecular positions of the reactant (even if extracted from product data), they reflect precisely those reaction steps that lead up to and include the first irreversible step. Consequently, they incorporate the initial reaction steps and their interpretation does not require consideration of the one *vs.* two-step case distinction as for PCE above.

**Figure 6:**
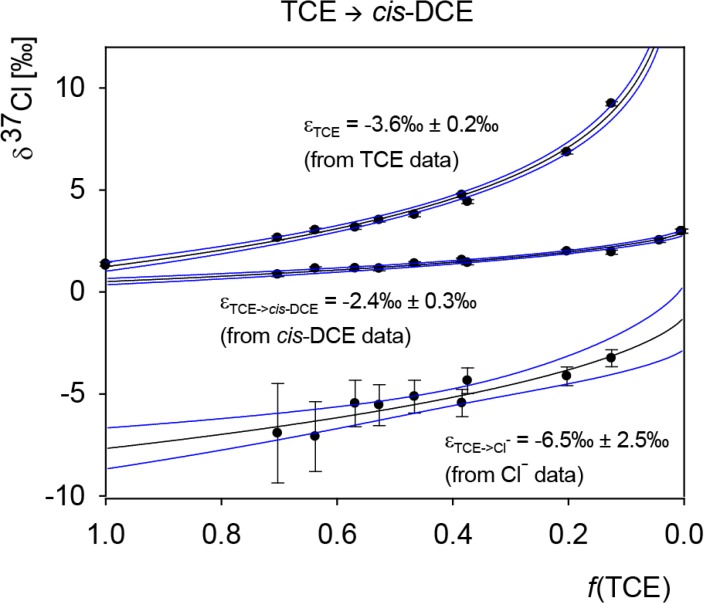
Chlorine isotope data of TCE degradation to *cis*-DCE and chloride by *Geobacter lovleyi* strain SZ including mathematical fits in Sigma Plot according to the presented mathematical approach. Error bars of individual data points indicate standard deviations; uncertainties of the regressions are 95% confidence intervals.

Figure 6 shows the fit to the obtained enrichment factor of TCE, which is the average of enrichment factors from each of its three chlorinated positions according to:

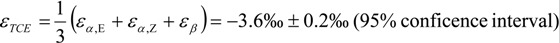
(33)


Since no 1,1-DCE is formed in *Geobacter lovleyi* cultures, the position Cl*_β_* in TCE does not react and will strictly represent a β-secondary isotope effect that is directly passed on to the formed *cis*-DCE with the enrichment factor ε_β_. In contrast, as discussed above, we considered the possibility that both α-chlorines may undergo C-Cl cleavage. 

The resultant product isotope enrichment for the formed *cis*-DCE was fitted in Sigma Plot according to Equation (25) derived previously to give

ε*_TCE->cis-DCE_* = −2.4‰ ± 0.3‰ (95% confidence interval)

Likewise, data on chloride were fitted according to Equation (24) to yield

ε*_TCE->Chloride_* = −6.5‰ ± 2.5‰ (95% confidence interval)

As derived above, our considerations show that these parameters reflect enrichment trends in the molecular positions of TCE according to

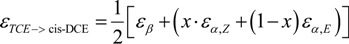
(23)

*ε_TCE_*_−> chloride_ = (*x·ε_α,E_* + (1 − *x*)·*ε_α,Z_*) (22)

Assuming that only Cl*_α,E_* is reacting (*x* = 1), the obtained enrichment trend of the formed chloride would strictly reflect a primary isotope effect with −6.5‰ ± 2.5‰. It follows that the formed *cis*-DCE would reflect only the average of secondary isotope effects, resulting in a remarkably large magnitude of secondary isotope effects with −2.4‰ ± 0.3‰. Therefore, this scenario of strictly one reactive position contrasts strongly with insight from PCE data, both regarding the absolute magnitude of primary isotope effects (too small) and secondary effects (too large) as well as the difference ε*_Diff_* between them (too small). It may be argued that small values (and small differences) could be attributable to commitment in enzyme catalysis, since our data relates to isotope effects on (V_max_/K_m_) rather than to elementary rate constants [34]. This would affect the interpretation of the TCE data, because the products *cis*-DCE and chloride reflect position-specific effects in the reactant TCE, and such reactant data are subject to masking [35]. The interpretation of the PCE data, in contrast, would not be affected, because product-curve intercepts reflect the differences in isotope effects in a situation of intramolecular competition, which is not subject to masking. While this could explain the small primary isotope effect of −6.5‰ ± 2.5‰ for TCE, it would be unable to rationalize the surprisingly large secondary isotope effect of −2.5‰ ± 0.3‰. Invoking commitment to catalysis would have to make this value even larger. Therefore, the assumption of only one reactive position appears to be questionable because it opposes the observation in PCE where a substantial difference in the magnitude of primary and secondary isotope effects was observed. 

A more consistent picture arises when the possibility of two reactive positions is considered, as encountered for any 1 > *x* > 0 in Figure 1. As illustrated in Figure 1, this scenario explains the relatively large isotope fractionation in the formed *cis*-DCE from a different angle, where a decrease in the difference between the isotope effects in cleaved chloride goes along with an increasingly even participation of both positions in the reaction. Therefore, the observation of the pronounced isotope effect of −2.5‰ ± 0.3‰ in the formation of *cis*-DCE with the relatively small difference to the isotope effect in the cleaved chloride of −6.5‰ ± 2.5‰ suggests that both positions react and primary and secondary chlorine isotope effects are reflected in both products. 

Our results therefore indicate that the two chlorine substituents in the α-positions are accessible for reductive dechlorination of TCE. In this context, the selective formation of *cis*-DCE as the only dichloroethene-isomer is an intriguing observation, because it requires that: (i) that two different positions react; (ii) different chlorovinyl-intermediates are formed, and (iii) these intermediates selectively react to *cis*-DCE despite their different stereochemistry. 

In previous mechanistic studies, the nucleophilic substitution of a chlorine substituent by the cobalt centre has been discussed as a potential pathway, leading to the formation of a dichlorovinyl-anion in the subsequent step of reduction [38,39]. Respective anionic intermediates have been investigated in a computational study by Nonnenberg *et al.*, which suggest that these intermeditates are not susceptible to interconversion [20], which would be necessary to explain the observed selectivity to form the *cis*-conformation. Based on our results, the scenario of a direct nucleophilic substitution can, therefore, be ruled out for reduction of TCE by *Geobacter lovleyi*.

**Scheme 4:**
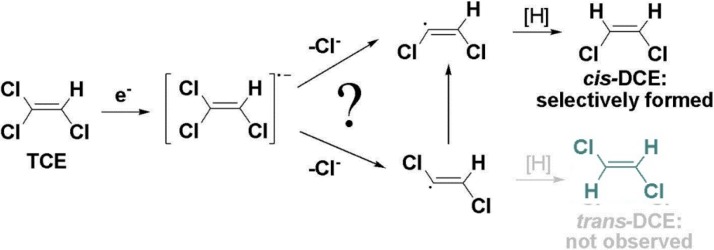
Mechanistic proposal for dechlorination of TCE via a single electron transfer with selective formation of *cis*-DCE. Adapted from Nonnenberg *et al.* [20].

Recent studies pointed out that radical intermediates are involved in reductive dechlorination with cobalamin as model system [15,17]. The behaviour of chlorovinyl-radical species was investigated in the computational study by Nonnenberg *et al.* [20]. From their calculations, the dichlorovinyl-radicals may indeed be susceptible to a selective interconversion from the *trans-* into the energetically preferred *cis*-conformation of DCE (Scheme 4). If this preference was further enforced by steric constraints at the catalytic site, this could explain an exclusive formation of *cis*-DCE observed in biodegradation experiments, which would otherwise even exceed thermodynamic predictions. According to the chlorine isotope effects from the presented biodegradation experiments of TCE, the involvement of radical intermediates therefore appears to be a possible pathway. 

**Scheme 5:**
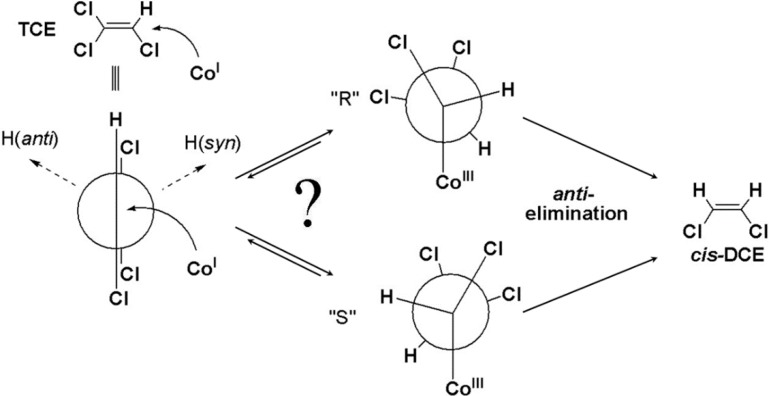
Mechanistic proposal for dechlorination of TCE via nucleophilic addition followed by *anti*-elimination with selective formation of *cis*-DCE. Adapted from Pratt and van der Donk [21].

Based on theoretical considerations, such a conformational change could also occur in a pathway of nucleophilic addition, where the addition of the cobalt centre of the corrinoid would lead to a change of hybridisation from sp^2^ to sp^3^ and create a freely rotating bond (Scheme 5). 

This intermediate was computed by Pratt and van der Donk [21], where they suggested that its minimum energy conformation may lead to a preferential formation of *cis*-DCE in the following step of *anti*-elimination. It is noteworthy that this scenario requires an extraordinary selectivity to engage this hypothesized conformation prior to the elimination reaction in order to produce exclusively *cis*-DCE. Furthermore, the pronounced chlorine isotope effects in TCE would indicate another exceptional feature of this scenario. In order to relate the obtained isotope effects to primary chlorine isotope effects, the elimination-step must be the rate-limiting step, while the first addition step would have to be reversible. The reversibility of the formation of the chlorinated alkylcobalt complex appears to be unlikely; however, since its formation is computed to have a notable driving force of −30.3 kcal/mol [14], and this reverse reaction would demand for the elimination of the hydrogen substituent. Therefore, the first step of the nucleophilic addition would not be predicted to be reversible.

With these considerations, the scenario of nucleophilic addition would combine several unusual attributes, including a strict conformational selectivity at an sp^3^ hybridized carbon centre and an unexpectedly large magnitude of secondary chlorine isotope effects. Nonetheless, this pathway cannot be strictly ruled out as a possible transformation pathway. 

## 6. Conclusions

With chlorine isotope effects from GC-IRMS measurements, our study brings forward a new perspective for investigating initial mechanisms of reductive chlorinated ethene dehalogenation. Mathematical equations accurately describe reactant and product isotope data, allow extracting position-specific chlorine isotope effect information and may serve as a benchmark for similar evaluations in the future. In reductive biotransformation of PCE, this evaluation allowed us to constrain the difference between primary and average secondary chlorine isotope effects to an unexpectedly large value of −16.3‰ ± 1.4‰ (standard error). This novel insight on primary and secondary chlorine isotope effects in chlorinated ethenes falls outside the range of typical chlorine isotope effects [40].

Our evaluation further allowed us to test whether one or two C-Cl bonds in TCE were reactive. In the first case (only one C-Cl position reacts), isotope values of chloride would be expected to exclusively reflect the primary isotope effect of the C-Cl bond cleavage, whereas isotope values of *cis*-DCE would reflect the secondary isotope effects in the remaining bonds. In contrast, in the second case both positions would take turns in reacting, they would end up in both products and the products would show a mixture of primary and secondary isotope effects. Their isotopic enrichment trends would, therefore, be more similar. Our data showed indeed only a relatively small difference between an average chlorine isotope effect of −6.5‰ ± 2.5‰ (95% confidence interval) in the C-Cl bond(s) from which chloride was formed, compared to an average effect of −2.5‰ ± 0.3‰ (95% confidence interval) in the C-Cl bonds that are precursors of *cis*-DCE. This small difference contrasts with the large difference between primary and secondary chlorine isotope effects observed in the PCE data. These findings suggest that two C-Cl bonds in TCE were reactive (case 2).

This insight, in turn, significantly constrained the mechanistic scenarios for the initial step in TCE reductive dehalogenation. Direct nucleophilic substitution via dichlorovinyl-anion intermediates could be ruled out for reduction of TCE by *Geobacter lovleyi*, whereas single electron transfer followed by radical formation, as well as nucleophilic addition followed by *anti*-elimination remain possible scenarios. 

**Figure 7:**
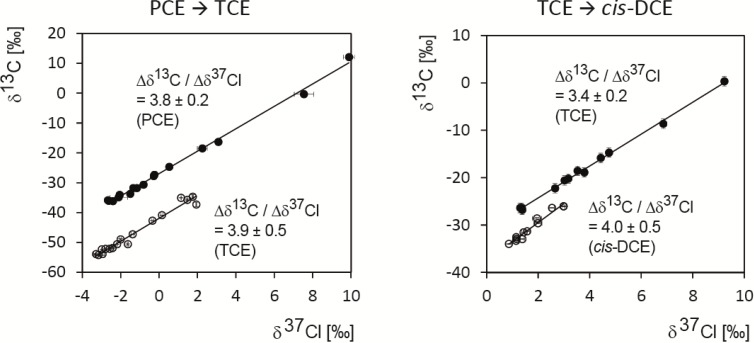
Dual element (carbon and chlorine) isotope plots for degradation of PCE toTCE by *Desulfitobacterium* sp. strain Viet1, and of TCE to *cis*-DCE by *Geobacter lovleyi* strain SZ. Error bars are standard deviations, uncertainties are 95% confidence intervals).

Finally, this insight can support current interpretations of dual element (C and Cl) isotope fractionation during TCE and PCE dehalogenation. Figure 7 shows the dual element isotope plots that are obtained when combining the carbon and chlorine data of the present study. 

The value of ∆δ^13^C_TCE_/∆δ^37^Cl_TCE_ = 3.4 ± 0.2 (95% confidence interval) during dehalogenation of TCE by *Geobacter lovleyi* is based on the same data reported in Cretnik *et al.* [29]. There, a remarkable similarity was observed compared to experiments with *Desulfitobacterium hafniense* and with vitamin B_12_. The present study complements this finding with underlying mechanistic insight. Taken together, our results suggest that this insight may also be of relevance for reductive dehalogenation by vitamin B_12_. In addition, our calculations can explain the different numerical values in the TCE degradation experiment when ∆δ^13^C/∆δ^37^Cl of TCE is compared to ∆δC^13^C/∆δ^37^Cl of *cis‑*DCE. While the slope for TCE is equal to ε*_TCE,carbon_*/ε*_TCE,chlorine_* [31], the slope for *cis*-DCE corresponds to ε*_TCE,carbon_*/ε*_TCE‑>cis‑DCE_*,_chlorine_. The observation that ε*_TCE->cis‑DCE,chlorine_* < ε*_TCE,chlorine_* (see above) therefore explains the steeper slope for the *cis*-DCE data. 

In the case of PCE, the dual element isotope slope of ∆δ^13^C*_PCE_*/∆δ^37^Cl*_PCE_* = 3.8 ± 0.2 (confidence interval) is, to our knowledge, the first value reported under defined conditions with a pure bacterial strain. The treatment of our study can explain in part why the value is greater compared to ∆δ^13^C*_TCE_*/∆δ^37^Cl*_TCE_* in the TCE experiment. In the compound-average chlorine ε*_PCE_* of the PCE experiment, the primary isotope effect in the reacting C-Cl bond is “diluted” with three secondary isotope effects according to Equation (32). In the TCE experiment, only two secondary isotope effects contribute so that the primary isotope effect is more strongly represented in ε*_TCE,chlorine_*. This, in turn, leads to a greater proportion of ε*_chlorine_*/ε*_carbon_* and a smaller value of the dual element isotope slope, since ε*_TCE,carbon_*/ε*_TCE,chlorine_* = ∆δ^13^C*_TCE_*/ ∆δ^37^Cl*_TCE_*. Finally, the value of ∆δ^13^C_PCE_/ ∆δ^37^Cl*_PCE_* = 3.8 ± 0.2 coincides with the range of 2.2 to 4.2 observed by Wigert *et al.* [28] in an enrichment culture from a contaminated site (calculated from their value ε*_chlorine_*/ε*_carbon_*= 0.35 ± 0.11). The combined results give a first indication of the range of ∆δ^13^C*_PCE_*/ ∆δ^37^Cl*_PCE_* that is expected for reductive dechlorination of PCE and TCE at contaminated sites. 

## Supplementary Materials

Supporting Information can be accessed at: http://www.mdpi.com/1420-3049/19/5/6450/s1.

A1 MATERIALS and METHODS: Experimental protocols for biodegradation of PCE with *Desulfitobacterium* Strain Viet-1 and of TCE with *Geobacter lovleyi* Strain SZ; for concentration measurements, stable carbon isotope analysis, stable chlorine isotope analysis; A2 DERIVATION of mathematical equations; A3 Figure S1: Visualisation of observable isotope trends in chloride with different contributions of primary and secondary isotope effects; A4 Figure S2: concentration *vs.* time curves of PCE degradation by *Desulfitobacterium* strain Viet1; Figure S3: concentration *vs.* time curves of TCE degradation by *Geobacter lovleyi* strain SZ. A5 original experimental data including δ^37^cl_cl_− and propagated errors; A6 equations to calcuate δ^37^cl_cl_− and propagated errors; A7 all fitting procedures and regression reports.
