# Supplementary material for: Chlorine Isotope Effects from Isotope Ratio Mass Spectrometry Suggest Intramolecular C-Cl Bond Competition in Trichloroethene (TCE) Reductive Dehalogenation
Source: Molecules. 2014 May 20;19(5):6450–73. doi: 10.3390/molecules19056450 (PMC6271514; doi:10.3390/molecules19056450)
Supplement: Supplementary file 1 [file molecules-19-06450-s001.zip › molecules-51800-supplementary.pdf]

Supporting Information for

Chlorine Isotope Effects from Isotope Ratio Mass Spectrometry Suggest Intramolecular C-Cl Bond Competition in Trichloroethene (TCE) Reductive Dehalogenation

Stefan Cretnik [1], Anat Bernstein [1], Orfan Shouakar-Stash [2], Frank Löffler [3,4,5] and Martin Elsner [1,*]

[1] Institute of Groundwater Ecology, Helmholtz Zentrum München, Ingolstädter Landstr. 1, 85764 Neuherberg, Germany; E-Mails: one_fiftyone@hotmail.com (S.C.); anatbern@bgu.ac.il (A.B.)

[2] Department of Earth and Environmental Sciences, University of Waterloo, Waterloo, ON N2L 3G1, Canada; E-Mail: orfan@uwaterloo.ca

[3] Department of Microbiology & Center for Environmental Biotechnology, University of Tennessee, Knoxville, TN 37996-2000, USA; E-Mail: frank.loeffler@utk.edu

[4] Department of Civil and Environmental Engineering, University of Tennessee, Knoxville, TN 37996-2000, USA

[5] Oak Ridge National Laboratory (UT-ORNL) Joint Institute for Biological Sciences (JIBS) and Bioscience Division, Oak Ridge National Laboratory, Oak Ridge, TN 37831, USA

* Author to whom correspondence should be addressed; E-Mail: martin.elsner@helmholtz-muenchen.de; Tel.: +49-89-3187-2565; Fax: +49-89-3187-3361.

Contents

A1. Materials and Methods S2

A1.1. Biodegradation of PCE with Desulfitobacterium Strain Viet-1 .... S2

A1.2. Biodegradation of TCE with Geobacter Lovleyi Strain SZ. S2

A1.3. Concentration Measurements S3

A1.4. Stable Carbon Isotope Analysis S3

A1.5. Stable Chlorine Isotope Analysis S3

A2. Equations S4

A2.1. Dechlorination Reactions with PCE. S4

A2.2. Dechlorination Reactions with TCE S5

A3. Different Contributions of Primary and Secondary Isotope Effects S7

A4. Concentration Profiles of Biodegradation Experiments S8

A5. Original Experimental Data Including $\delta^{37}Cl_{Cl^-}$ and Propagated Errors S8

A6. Equations to Calcuate $\delta^{37}Cl_{Cl^-}$ and Propagated Errors S8

A6.1. TCE Experiment. S8

A6.2. PCE Experiment.. .... S9

A7. Fitting Procedures and Regression Reports S9

A7.1. Regressions of Figure 2 .... S9

A7.2. Regressions of Figure 3 .... S17

A7.3. Regressions of Figure 6 .... S22

Total Number of Pages: 27 + one additional Excel Document (A5)

A1. Materials and Methods

A1.1. Biodegradation of PCE with Desulfitobacterium Strain Viet-1

Biodegradation experiments of PCE were carried out using the microbial strain *Desulfitobacterium* strain VIET-1, which reductively dechlorinates PCE to the final product TCE. It was gratefully provided by Frank Loeffler and his collection of Microorganisms at University of Tennessee and it was cultured according to DSMZ instructions, medium 720, with PCE as the electron acceptor. The growth medium for the experiment was prepared in glass bottles (250 mL), equipped with Mininert valves (Supelco, Bellefonte, PA, USA), and filled with 150 mL of medium, leaving a headspace of 40%. The bottles were amended with 10 µL of neat PCE and constantly shaken on a horizontal shaker at 120 rpm for four days. Inoculation was carried out by adding 20 mL of active culture, which was previously grown in a similar medium. To eliminate carry-over of the degradation product (TCE) to the fresh medium, the media with the culture that was used for inoculation was flushed with N_2/CO_2 gas stream (80%/20%) for 5 h prior transferring to the fresh medium. A complete removal of chloroethenes after degassing was controlled by GC-FID measurements. This procedure was followed for three biological replicates. Abiotic control batches were prepared similarly, but without inoculation of the active culture. Sampling was carried out 20 min after inoculation for the initial sample, and at given time points along the degradation. A total sample volume of 7 mL was taken with a glass syringe (Hamilton, ON, Canada), which was distributed in portions of 1 mL each into 7 amber vials with an active volume of 1.6 mL. In order to stop biological activity, the vials were spiked with 50 µL of NaOH (1 M) and closed with PTFE-lined screw caps. All vials were frozen upside down for subsequent isotope analysis, except one vial, which was used immediately for concentration analysis.

A1.2. Biodegradation of TCE with Geobacter Lovleyi Strain SZ

Biodegradation experiments of TCE were carried out using the microbial strain *Geobacter lovleyi* strain SZ, purchased from the German Collection of Microorganisms and Cell Cultures (DSMZ, Braunschweig, Germany). This strain reductively dechlorinates TCE to the final product *cis*-DCE. A growth medium was prepared according to DSMZ instructions, medium 732, with the exception that neither hexadecane nor perchloroethylene was added to the medium. The growth medium for the experiment was prepared in glass bottles (250 mL), equipped with Mininert valves (Supelco, Bellefonte, PA, USA), and filled with 150 mL of medium, leaving a headspace of 40%. The bottles were amended with 10 µL of neat TCE and constantly shaken on a horizontal shaker at 120 rpm for four days. Inoculation was carried out by adding 14 mL of active culture, which was previously grown in a similar medium. To eliminate carry-over of the degradation product (*cis*-DCE) to the fresh medium, the media with the culture that was used for inoculation was flushed with N_2/CO_2 gas stream (80%/20%) for 5 h prior transferring to the fresh medium. A complete removal of chloroethenes after degassing was controlled by GC-FID measurements. This procedure was followed for three biological replicates. Abiotic control batches were prepared similarly, but without inoculation of the active culture. Sampling was carried out 20 min after inoculation for the initial sample, and at given time points along the degradation. A total sample volume of 7 mL was taken with a glass syringe (Hamilton, ON, Canada), which was distributed in portions of 1 mL each into 7 amber vials with an

active volume of 1.6 mL. In order to stop biological activity, the vials were spiked with 50 µL of NaOH (1 M) and closed with PTFE-lined screw caps. All vials were frozen upside down for subsequent isotope analysis, except one vial, which was used immediately for concentration analysis.

A1.3. Concentration Measurements

PCE, TCE and *cis*-DCE concentrations in the biodegradation experiments were measured by a gas chromatograph equipped with flame ionization detector (GC-FID, Hewlett Packard 5890 Series II) equipped with a 30 m VOCOL column (Supelco, Bellefonte, PA, USA) 0.25 mm inner diameter, with a film thickness of 1.5 µm and operated with nitrogen as carrier gas at 1.6 mL/min. Automated headspace injections of 1 mL from 10 mL headspace vials were carried out using a Pal™ autosampler (CTC Analytics), and an injector temperature on the GC of 200 °C. Calibrations were performed along each measurement using solutions of the chloroethenes with concentrations between 4.0 and 383.9 mg/L. The resulting total relative error in concentrations was estimated as ±10%.

A1.4. Stable Carbon Isotope Analysis

Compound Specific Isotope Analysis (CSIA) for carbon was conducted by injection of headspace samples on a GC-IRMS system (Thermo Fisher Scientific, Waltham, MA, USA) consisting of a Trace GC with a Pal™ autosampler (CTC Analytics), coupled to a MAT 253 IRMS through a GC/C III combustion interface. The gas chromatograph was equipped with a 30 m VOCOL column (Supelco, Bellefonte, PA, USA), 0.25 mm inner diameter, with a film thickness of 1.5 µm and operated with He carrier gas at 1.4 mL/min. The GC program started at 85 °C (8 min) and increased at 60 °C/min to 205 °C (1 min). Internal standards of PCE, TCE and *cis*-DCE were used along the measurements. The analytical uncertainty 2σ of carbon isotope analysis was ±0.5‰.

A1.5. Stable Chlorine Isotope Analysis

Chlorine isotope analysis of PCE TCE, and *cis*-DCE was performed according to a method adapted from Shouakar-Stash *et al.* (2006). PCE TCE, and *cis*-DCE are transferred from a Trace-GC (Thermo Scientific, Waltham, MA, USA) to the MAT 253 IRMS through the He carrier stream, where the chloroethenes are ionized and fragmented for isotope ratio measurements. The measurements were conducted at masses m/z = 94, 96 for PCE, m/z = 95, 97 for TCE, m/z = 96, 98 for *cis*-DCE. The gas chromatograph was equipped with a 30 m VOCOL column (Supelco, Bellefonte, PA, USA) with 0.25 mm inner diameter, a film thickness of 1.5 µm and operated with a He carrier gas at 1.4 mL/min. The GC program used started at 50 °C (7 min), increasing at 60 °C/min to 70 °C (2.70 min) and at 80 °C/min to 140 °C (0.10 min). External standards were measured daily for calibration of δ^{37}Cl values according to Bernstein *et al.* Briefly, a reference gas of each target analyte is introduced via a dual inlet system. In order to enable isotope measurements of two chlorinated ethenes in one run, the chlorinated ethene with the shorter retention time was introduced at the beginning of each run from one bellow of the dual inlet, while at the end of each run the chlorinated ethene with the longer retention time was introduced from the other bellow. The conversion to delta values relative to the international reference Standard Mean Ocean Chloride (SMOC) was performed by an external two-point calibration analysing

chloroethene-standards as previously characterized in the Department of Earth Sciences, University of Waterloo. Each of these standards was added in triplicates before, during and at the end of each sequence, in order to calibrate the obtained values of the samples with respect to SMOC. The analytical uncertainty 2σ of chlorine isotopic measurements was ±0.2‰.

A2. Equations

The following considerations are based on the one hand on Rayleigh equation, as it is well established to express enrichment factors ε for a certain element E in a substrate along a certain progress of reaction *f* according to Equation (9) in the manuscript with

$$\delta^h E = \delta^h E_0 + \varepsilon \cdot \ln f \tag{S1}$$

On the other hand, an isotopic mass balance can be performed for any reaction in a closed system. Here, the reactant contains m_S atoms of element *E* in its structure. $\delta^h E_0$ is the original reactant isotope ratio, whereas $\delta^h E$ is the ratio when reaction has occurred so that only a fraction *f* of reactant remains. A fraction of (*1* − *f*) has then been converted to one or more (up to n) products; m_i is the number of atoms of *E* inside the structure of product *i*, $\delta^h E_{P,i}$ is the respective product's isotope value. In the Manuscript, the respective relationship is given with Equation (11) with

$$\sum_{i=1}^{n} m_i \cdot \delta^h E_{P,i} = \frac{m_S \cdot \delta^h E_0 - m_S \cdot f \cdot \delta^h E}{(1-f)} \tag{S2}$$

A2.1. Dechlorination Reactions with PCE

In the case of PCE, molecular positions are chemically equivalent so that the same chlorine atoms may potentially end up in TCE or Cl^-. Isotopes then partition according to the kinetic isotope effects associated with the formation of either product, $\alpha_i = 1/KIE_i$. As a consequence, in both cases their isotope ratios relate according to

$$\frac{R_{P_1}}{R_{P_2}} = \frac{\alpha_1}{\alpha_2} \tag{S3}$$

This can be expressed in the delta notation:

$$\frac{\delta^{37}Cl_{P_1} + 1}{\delta^{37}Cl_{P_2} + 1} = \frac{\alpha_1}{\alpha_2} = \alpha_{Diff} \tag{S4}$$

with α_{Diff} expressing the ratio between the primary isotope effect (in the formation of Cl^-) and the average secondary isotope effects (in the three molecular positions which become TCE). This equation can be rearranged and simplified according to

$$\delta^{37}Cl_{P_1} + 1 = \alpha_{Diff}(\delta^{37}Cl_{P_2} + 1) \rightarrow \delta^{37}Cl_{P_1} - \underbrace{\alpha_{Diff}}_{\approx 1} \cdot \delta^{37}Cl_{P_2} = \alpha_{Diff} - 1 \tag{S5}$$

$$\rightarrow \delta^{37}Cl_{P_1} - \delta^{37}Cl_{P_2} = \alpha_{Diff} - 1 = \varepsilon_{Diff} \tag{S6}$$

This means the difference between primary and secondary isotope effects ε_{Diff} is directly obtained from product isotope values, because chlorine isotope ratios of Cl^- and TCE are always by ε_{Diff} apart.

This can be combined with the isotopic mass balance for the case of PCE degradation to TCE according to

$$\delta^{37}Cl_{0,PCE} = f \cdot \delta^{37}Cl_{PCE} + \frac{1}{4}(1-f)\delta^{37}Cl_{Cl^-} + \frac{3}{4}(1-f)\cdot\delta^{37}Cl_{TCE} \tag{S7}$$

Equation (17) in the manuscript is here expressed in Equation (S8) with

$$\begin{gathered}\delta^{37}Cl_{TCE} - \delta^{37}Cl_{Cl^-} = \varepsilon_{Diff} \\ \rightarrow \delta^{37}Cl_{TCE} = \varepsilon_{Diff} + \delta^{37}Cl_{Cl^-} \text{ and } \delta^{37}Cl_{Cl^-} = \delta^{37}Cl_{TCE} - \varepsilon_{Diff}\end{gathered} \tag{S8}$$

When Equations (S7) and (S8) is combined, we can resolve the isotope signatures individually for chloride according to

$$\delta^{37}Cl_{0,PCE} = f \cdot \delta^{37}Cl_{PCE} + \frac{1}{4}(1-f)\delta^{37}Cl_{Cl^-} + \frac{3}{4}(1-f)\cdot\varepsilon_{Diff} + \frac{3}{4}(1-f)\cdot\delta^{37}Cl_{Cl^-}$$

Together with the Rayleigh enrichment trend for PCE, the isotope trends of the formed chloride can be expressed with

$$\delta^{37}Cl_{Cl^-} = \underbrace{\delta^{37}Cl_{0,PCE} - \frac{3}{4}\varepsilon_{Diff}}_{constant: K_{Cl^-}} - \varepsilon_{chlorine} \cdot \frac{f \cdot \ln f}{(1-f)} \tag{S9}$$

A similar procedure can be followed in order to resolve Equations (S7) and (S8) towards TCE, and we obtain an expression to model the enrichment trend of TCE with

$$\delta^{37}Cl_{TCE} = \underbrace{\delta^{37}Cl_{0,PCE} + \frac{1}{4}\varepsilon_{Diff}}_{constant: K_{TCE}} - \varepsilon_{chlorine} \cdot \frac{f \cdot \ln f}{(1-f)} \tag{S10}$$

The equations to Equations (S9) and (S10) are equal to Equations (18) and (19) from the manuscript, which were used for mathematical modeling of product isotope enrichment of PCE.

A2.2. Dechlorination Reactions with TCE

In the case of TCE, molecular positions are chemically distinguishable, and a structural preference is present in the α-position according to the selective formation of *cis*-DCE so that the same chlorine atoms may potentially end up in TCE or Cl^-. It is, then, of interest to which percentage of $Cl_{\alpha,E}$ and $Cl_{\alpha,Z}$ react to form the cleaved chloride. A factor x can be introduced to express this as

x = percentage that reacts from $Cl_{\alpha,E}$ to $Cl^-_{\alpha,2}$

$(1 - x)$ = percentage that reacts from $Cl_{\alpha,Z}$ to $Cl^-_{\alpha,2}$

with an $x = 1$ the reaction would follow a position-specific cleavage, while any $1 > x > 0$ would reflect a case where two positions are involved. For each of the three chlorinated positions the mass balance can be raised individually in an extension of the equations describing the isotopic mass balance

$$\delta^{37}Cl_{0,\alpha,E} = f\cdot\delta^{37}Cl_{\alpha,E} + x\cdot(1-f)\cdot\delta^{37}Cl^{from\alpha,E}_{Cl^-_{\alpha,2}} + (1-x)(1-f)\cdot\delta^{37}Cl^{from\alpha,E}_{cDCE,\alpha,1} \qquad \text{(S11)}$$

$$\delta^{37}Cl_{0,\alpha,Z} = f\cdot\delta^{37}Cl_{\alpha,Z} + (1-x)\cdot(1-f)\cdot\delta^{37}Cl^{from\alpha,Z}_{Cl^-_{\alpha,2}} + x\cdot(1-f)\cdot\delta^{37}Cl^{from\alpha,Z}_{cDCE,\alpha,1} \qquad \text{(S12)}$$

$$\delta^{37}Cl_{0,\beta} = f\cdot\delta^{37}Cl_{\beta} + (1-f)\cdot\delta^{37}Cl_{cDCE,\beta} \qquad \text{(S13)}$$

For the two individual reacting positions, the difference in their isotope signatures reflect the difference of position specific enrichment factors for the case of a primary isotope effect with the formation of chloride, or a secondary isotope effect with the formation of cis-DCE. This difference of enrichment factors have to be treated separately for $Cl_{\alpha,E}$ and $Cl_{\alpha,Z}$, according to

$$\delta^{37}Cl^{from\alpha,E}_{Cl^-_{\alpha,2}} - \delta^{37}Cl^{from\alpha,E}_{cDCE,\alpha,1} = \varepsilon_{Diff,\alpha,E} \qquad \text{(S14)}$$

$$\delta^{37}Cl^{from\alpha,Z}_{Cl^-_{\alpha,2}} - \delta^{37}Cl^{from\alpha,Z}_{cDCE,\alpha,1} = \varepsilon_{Diff,\alpha,Z} \qquad \text{(S15)}$$

These differences in fractionation factors can be included in the position specific mass balance to give

$$\delta^{37}Cl^{from\alpha,E}_{Cl^-_{\alpha,2}} = \delta^{37}Cl_{0,\alpha,E} + (1-x)\cdot\varepsilon_{Diff,\alpha,E} - \varepsilon_{\alpha,E}\frac{f\ln f}{(1-f)} \qquad \text{(S16)}$$

$$\delta^{37}Cl^{from\alpha,Z}_{Cl^-_{\alpha,2}} = \delta^{37}Cl_{0,\alpha,Z} + x\cdot\varepsilon_{Diff,\alpha,Z} - \varepsilon_{\alpha,Z}\frac{f\ln f}{(1-f)} \qquad \text{(S17)}$$

$$\delta^{37}Cl^{from\alpha,E}_{cDCE,\alpha,1} = \delta^{37}Cl_{0,\alpha,E} - x\cdot\varepsilon_{Diff,\alpha,E} - \varepsilon_{\alpha,E}\frac{f\ln f}{(1-f)} \qquad \text{(S18)}$$

$$\delta^{37}Cl^{from\alpha,Z}_{cDCE,\alpha,1} = \delta^{37}Cl_{0,\alpha,Z} - (1-x)\cdot\varepsilon_{Diff,\alpha,Z} - \varepsilon_{\alpha,Z}\frac{f\ln f}{(1-f)} \qquad \text{(S19)}$$

$$\delta^{37}Cl_{cDCE,\beta} = \delta^{37}Cl_{0,\beta} - \varepsilon_{\beta}\frac{f\ln f}{(1-f)} \qquad \text{(S20)}$$

The isotopic mass balance can now be set up for the cleaved chloride according to

$$\begin{aligned}\delta^{37}Cl_{Cl^-_{\alpha,2}} &= \left(x\cdot\delta^{37}Cl^{from\alpha,E}_{Cl^-_{\alpha,2}} + (1-x)\cdot\delta^{37}Cl^{from\alpha,Z}_{Cl^-_{\alpha,2}}\right)\\ &= \left(x\cdot\delta^{37}Cl_{0,\alpha,E} + x(1-x)\cdot\varepsilon_{Diff,\alpha,E} - x\varepsilon_{\alpha,E}\frac{f\ln f}{(1-f)} + (1-x)\delta^{37}Cl_{0,\alpha,Z} + (1-x)\cdot x\cdot\varepsilon_{Diff,\alpha,Z} - (1-x)\cdot\varepsilon_{\alpha,Z}\frac{f\ln f}{(1-f)}\right)\\ &= \underbrace{\left(x\cdot\delta^{37}Cl_{0,\alpha,E} + (1-x)\delta^{37}Cl_{0,\alpha,Z} + x(1-x)\cdot\left[\varepsilon_{Diff,\alpha,E} + \varepsilon_{Diff,\alpha,Z}\right]\right)}_{constant:K_{Cl^-}} - \underbrace{\left[x\cdot\left(\varepsilon_{\alpha,E} - \varepsilon_{\alpha,Z}\right) + \varepsilon_{\alpha,Z}\right]}_{\varepsilon_{TCE->\text{chloride}}}\frac{f\ln f}{(1-f)}\end{aligned} \qquad \text{(S21)}$$

The enrichment factor can be extracted here in order to reflect Equation (22) from the manuscript

$$\varepsilon_{TCE->\text{chloride}} = \left(x\cdot\varepsilon_{\alpha,E} + (1-x)\cdot\varepsilon_{\alpha,Z}\right) \qquad \text{(S22)}$$

In the interpretation of our experiments, isotope data of chloride was therefore modeled with

$$\delta^{37}Cl_{Cl^-} = K_{Cl^-} - \varepsilon_{TCE->\text{chloride}}\frac{f\ln f}{(1-f)} \qquad \text{(S23)}$$

Also in the case of *cis*-DCE, an isotopic mass balance could be set up according to

$$\delta^{37}Cl_{cDCE} = \frac{1}{2}\delta^{37}Cl_{\beta} + \frac{1}{2}\left[(1-x)\cdot\delta^{37}Cl_{cDCE,\alpha,1}^{from\alpha,E} + x\cdot\delta^{37}Cl_{cDCE,\alpha,1}^{from\alpha,Z}\right]$$
$$= \frac{1}{2}\left(\delta^{37}Cl_{0,\beta} - \varepsilon_{\beta}\frac{f\ln f}{(1-f)}\right)$$
$$+\frac{1}{2}\left[(1-x)\left(\delta^{37}Cl_{0,\alpha,E} - x\cdot\varepsilon_{Diff,\alpha,E} - \varepsilon_{\alpha,E}\frac{f\ln f}{(1-f)}\right) + x\left(\delta^{37}Cl_{0,\alpha,Z} - (1-x)\cdot\varepsilon_{Diff,\alpha,Z} - \varepsilon_{\alpha,Z}\frac{f\ln f}{(1-f)}\right)\right] \quad (S24)$$
$$= \underbrace{\frac{1}{2}\left(\delta^{37}Cl_{0,\beta} + \frac{(1-x)\cdot\delta^{37}Cl_{0,\alpha,E} + x\cdot\delta^{37}Cl_{0,\alpha,Z}}{2}\right) + \frac{1}{2}x\cdot(1-x)\left[\varepsilon_{Diff,\alpha,E} - \varepsilon_{Diff,\alpha,Z}\right]}_{constant:K_{cDCE}} - \underbrace{\frac{1}{2}\left[\varepsilon_{\beta} + x\left(\varepsilon_{\alpha,Z} - \varepsilon_{\alpha,E}\right) + \varepsilon_{\alpha,E}\right]}_{\varepsilon_{TCE\text{-->}\,\text{cis-DCE}}}\frac{f\ln f}{(1-f)}$$

The enrichment factor can be extracted here in order to reflect Equation (23) from the manuscript

$$\varepsilon_{TCE\text{-->}\,\text{cis-DCE}} = \frac{1}{2}\left[\varepsilon_{\beta} + \left(x\cdot\left(\varepsilon_{\alpha,Z} - \varepsilon_{\alpha,E}\right) + \varepsilon_{\alpha,E}\right)\right] = \frac{1}{2}\left[\varepsilon_{\beta} + \left(x\cdot\varepsilon_{\alpha,Z} + (1-x)\varepsilon_{\alpha,E}\right)\right] \quad (S25)$$

Modeling of the obtained isotope data of cis-DCE from our experiments is therefore possible with

$$\delta^{37}Cl_{cDCE} = K_{cDCE} - \varepsilon_{TCE\text{-->}\,\text{cis-DCE}}\frac{f\ln f}{(1-f)} \quad (S26)$$

A3. Different Contributions of Primary and Secondary Isotope Effects

A different numerical scenario is visualized here, in order to show how different contributions in the α-positions depend on x, by accounting for different primary and secondary chlorine isotope effects for the individual positions. The exemplary numeric values here were $\varepsilon_{\alpha,E,primary} = -10‰$; $\varepsilon_{\alpha,E,secondary} = -3‰$; $\varepsilon_{\alpha,Z,primary} = -8‰$; $\varepsilon_{\alpha,Z,secondary} = -1‰$. The representation shows a similar qualitative trend, where (i) ε_{α} is stronger in the position from which more chloride is formed; and (ii) in addition, more atoms of this position are passed on to chloride so that product curve of chloride more strongly reflects this higher enrichment trend. The opposite trend can be observed in the product curve of *cis*-DCE.

Figure S1. Visualisation of Figure 1 with different contributions of primary and secondary isotope effects.

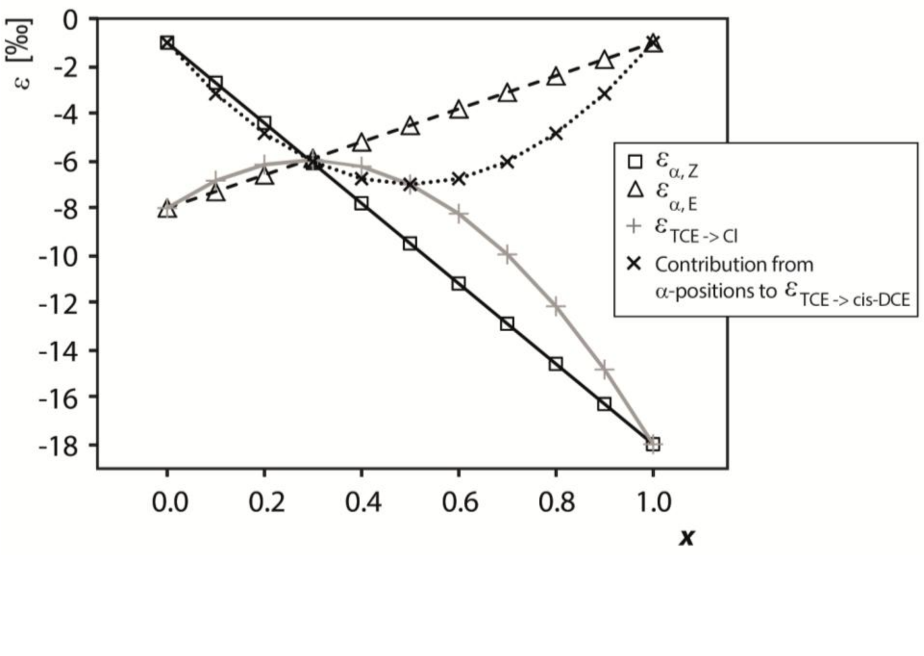

A4. Concentration Profiles of Biodegradation Experiments

Figure S2. PCE degradation by *Desulfitobacterium* strain Viet1.

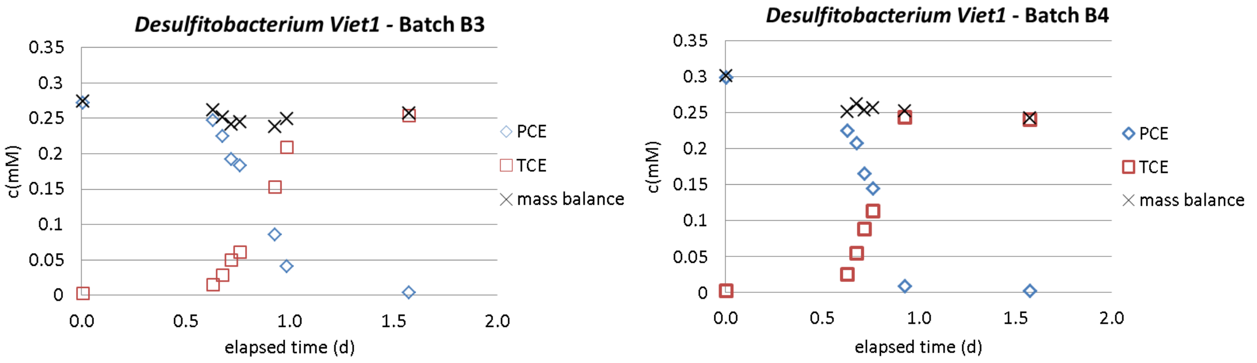

Figure S3. TCE degradation by *Geobacter lovleyi* strain SZ.

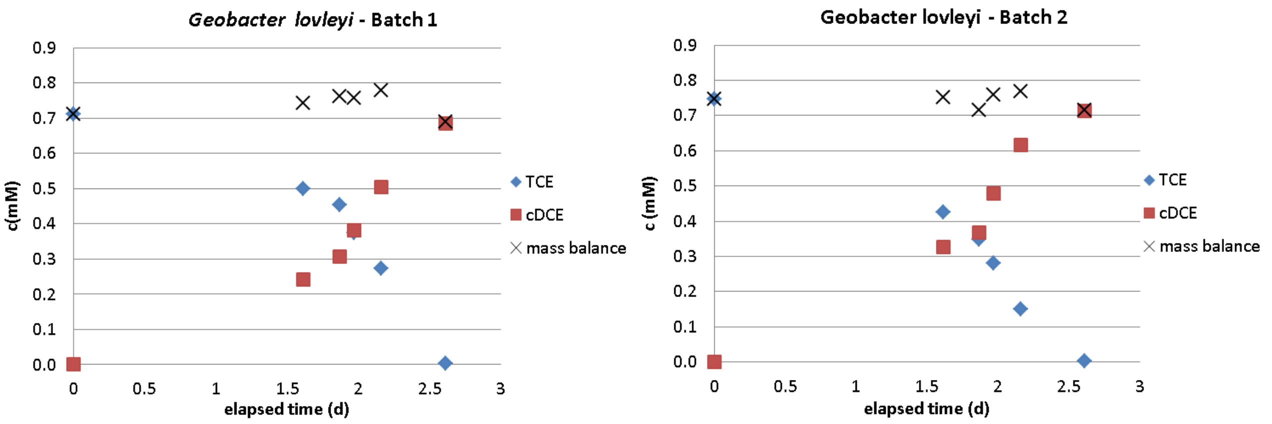

A5. Original Experimental Data Including $\delta^{37}Cl_{Cl^-}$ and Propagated Errors

See attached Excel Document.

A6. Equations to Calcuate $\delta^{37}Cl_{Cl^-}$ and Propagated Errors

A6.1. TCE Experiment

$$\delta^{37}Cl_{Cl^-} = \left[3\cdot\delta^{37}Cl_{0,TCE} - 3\cdot\delta^{37}Cl_{TCE}\cdot f - 2\cdot\delta^{37}Cl_{cis-DCE}\cdot(1-f)\right]/(1-f)$$

Error in the parameter as a result of error propagation:

$$\Delta\left(\delta^{37}Cl_{Cl^-}\right) = \sqrt{\begin{array}{l}\left[\partial\left(\delta^{37}Cl_{Cl^-}\right)/\partial\left(\delta^{37}Cl_{0,TCE}\right)\right]^2\cdot\left[\Delta\left(\delta^{37}Cl_{0,TCE}\right)\right]^2 \\ +\left[\partial\left(\delta^{37}Cl_{Cl^-}\right)/\partial\left(\delta^{37}Cl_{TCE}\right)\right]^2\cdot\left[\Delta\left(\delta^{37}Cl_{TCE}\right)\right]^2 \\ +\left[\partial\left(\delta^{37}Cl_{Cl^-}\right)/\partial\left(\delta^{37}Cl_{cis-DCE}\right)\right]^2\cdot\left[\Delta\left(\delta^{37}Cl_{cis-DCE}\right)\right]^2 \\ +\left[\partial\left(\delta^{37}Cl_{Cl^-}\right)/\partial f\right]^2\cdot\left[\Delta f\right]^2\end{array}}$$

$$\partial\left(\delta^{37}Cl_{Cl^-}\right)/\partial\left(\delta^{37}Cl_{0,TCE}\right)=3/(1-f)$$

$$\partial\left(\delta^{37}Cl_{Cl^-}\right)/\partial\left(\delta^{37}Cl_{TCE}\right)=-3\cdot f/(1-f)$$

$$\partial\left(\delta^{37}Cl_{Cl^-}\right)/\partial\left(\delta^{37}Cl_{cis-DCE}\right)=-2$$

$$\begin{aligned}\partial\left(\delta^{37}Cl_{Cl^-}\right)/\partial f =& \left[-3\cdot\delta^{37}Cl_{TCE}+2\cdot\delta^{37}Cl_{cis-DCE}\right]/(1-f)\\ &+\left[3\cdot\delta^{37}Cl_{0,TCE}-3\cdot\delta^{37}Cl_{TCE}\cdot f-2\cdot\delta^{37}Cl_{cis-DCE}\cdot(1-f)\right]/(1-f)^2\end{aligned}$$

A6.2. PCE Experiment

$$\delta^{37}Cl_{Cl^-}=\left[4\cdot\delta^{37}Cl_{0,PCE}-4\cdot\delta^{37}Cl_{PCE}\cdot f-3\cdot\delta^{37}Cl_{TCE}\cdot(1-f)\right]/(1-f)$$

Error in the parameter as a result of error propagation:

$$\Delta\left(\delta^{37}Cl_{Cl^-}\right)=\sqrt{\begin{aligned}&\left[\partial\left(\delta^{37}Cl_{Cl^-}\right)/\partial\left(\delta^{37}Cl_{0,PCE}\right)\right]^2\cdot\left[\Delta\left(\delta^{37}Cl_{0,PCE}\right)\right]^2\\&+\left[\partial\left(\delta^{37}Cl_{Cl^-}\right)/\partial\left(\delta^{37}Cl_{PCE}\right)\right]^2\cdot\left[\Delta\left(\delta^{37}Cl_{PCE}\right)\right]^2\\&+\left[\partial\left(\delta^{37}Cl_{Cl^-}\right)/\partial\left(\delta^{37}Cl_{TCE}\right)\right]^2\cdot\left[\Delta\left(\delta^{37}Cl_{TCE}\right)\right]^2\\&+\left[\partial\left(\delta^{37}Cl_{Cl^-}\right)/\partial f\right]^2\cdot\left[\Delta f\right]^2\end{aligned}}$$

$$\partial\left(\delta^{37}Cl_{Cl^-}\right)/\partial\left(\delta^{37}Cl_{0,PCE}\right)=4/(1-f)$$

$$\partial\left(\delta^{37}Cl_{Cl^-}\right)/\partial\left(\delta^{37}Cl_{PCE}\right)=-4\cdot f/(1-f)$$

$$\partial\left(\delta^{37}Cl_{Cl^-}\right)/\partial\left(\delta^{37}Cl_{TCE}\right)=-3$$

$$\begin{aligned}\partial\left(\delta^{37}Cl_{Cl^-}\right)/\partial f =& \left[-4\cdot\delta^{37}Cl_{PCE}+3\cdot\delta^{37}Cl_{TCE}\right]/(1-f)\\ &+\left[4\cdot\delta^{37}Cl_{0,PCE}-4\cdot\delta^{37}Cl_{PCE}\cdot f-3\cdot\delta^{37}Cl_{TCE}\cdot(1-f)\right]/(1-f)^2\end{aligned}$$

A7. Fitting Procedures and Regression Reports

Fits were conducted with non-liner Regressions in Sigma Plot 12.0 for Windows. Reports of all regressions are given below.

A7.1. Regressions of Figure 2

Fit of d13C Data of PCE in PCE Experiment

f = a + ((k) × ln(x))

**R**	**Rsqr**	**Adj Rsqr**	**Standard Error of Estimate**
0.997	0.993	0.993	1.158

	**Coefficient**	**Std. Error**	**t**	***p***
k	−18.960	0.412	−45.995	<0.0001
a	−37.407	0.389	−96.244	<0.0001

Confidence Intervals:

	**Coefficient**	**95% Conf-L**	**95% Conf-U**
k	−18.960	−19.844	−18.076
a	−37.407	−38.241	−36.573

Analysis of Variance:

**DF**		**SS**	**MS**
Regression	2	13,224.176	6612.088
Residual	14	18.774	1.341
Total	16	13,242.951	827.684

Corrected for the mean of the observations:

	**DF**	**SS**	**MS**	**F**	***p***
Regression	1	2836.974	2836.974	2115.513	<0.0001
Residual	14	18.774	1.341		
Total	15	2855.748	190.383		

Statistical Tests:

Normality Test (Shapiro-Wilk) Passed (p = 0.0771)

W Statistic = 0.8989 Significance Level = 0.0500

Constant Variance Test Passed (p = 0.2567)

95% Confidence:

**Row**	**Predicted**	**95% Conf-L**	**95% Conf-U**
1	−37.407	−38.241	−36.573
2	−35.878	−36.666	−35.090
3	−34.041	−34.779	−33.303
4	−31.093	−31.767	−30.419
5	−30.223	−30.882	−29.564
6	−15.242	−16.026	−14.459
7	−0.980	−2.281	0.320
9	−37.407	−38.241	−36.573
10	−32.343	−33.042	−31.645

11	−30.753	−31.421	−30.085
12	−26.434	−27.056	−25.811
13	−23.869	−24.494	−23.243
16	−37.407	−38.241	−36.573
17	−28.031	−28.663	−27.399
18	−17.410	−18.136	−16.684
19	10.848	9.044	12.652

Fit Equation Description:

[Variables]

x = col(1)
DepVar0 = col(2)
[Parameters]
k = 1 ' {{previous: −18.9603}}
a = 1 ' {{previous: −37.4069}}

[Equation]

f = a + ((k) × ln(x))
fit f to DepVar0

[Constraints]

[Options]

tolerance = 1e-010
stepsize = 1
iterations = 200

Number of Iterations Performed = 8

Fit of d13C Data of TCE in PCE Experiment

f = a − ((k) × (x × ln(x))/(1 − x))

**R**	**Rsqr**	**Adj Rsqr**	**Standard Error of Estimate**
0.983	0.966	0.963	1.396

	**Coefficient**	**Std. Error**	**t**	***p***
k	−21.138	1.029	−20.546	<0.0001
a	−35.072	0.659	−53.180	<0.0001

Confidence Intervals:

	**Coefficient**	**95% Conf-L**	**95% Conf-U**
k	−21.138	−23.331	−18.945
a	−35.072	−36.478	−33.667

Analysis of Variance:

	**DF**	**SS**	**MS**
Regression	2	37899.491	18949.746
Residual	15	29.221	1.948
Total	17	37928.712	2231.101

Corrected for the mean of the observations:

	**DF**	**SS**	**MS**	**F**	***p***
Regression	1	822.353	822.353	422.140	<0.0001
Residual	15	29.221	1.948		
Total	16	851.574	53.223		

Statistical Tests:

Normality Test (Shapiro-Wilk) Passed (p = 0.1999)

W Statistic = 0.9278 Significance Level = 0.0500

Constant Variance Test Passed (p = 0.2930)

95% Confidence:

**Row**	**Predicted**	**95% Conf-L**	**95% Conf-U**
2	−55.370	−56.523	−54.217
3	−54.390	−55.465	−53.314
4	−52.886	−53.851	−51.920
5	−52.458	−53.395	−51.522
6	−46.209	−46.933	−45.486
7	−42.039	−42.908	−41.171
8	−36.341	−37.636	−35.047
10	−53.513	−54.523	−52.504
11	−52.718	−53.672	−51.764
12	−50.680	−51.512	−49.849
13	−49.555	−50.334	−48.775
14	−37.303	−38.516	−36.090
15	−36.029	−37.350	−34.707
17	−51.413	−52.285	−50.542

18	−46.988	−47.710	−46.266
19	−39.653	−40.681	−38.626
20	−36.375	−37.667	−35.084

Fit Equation Description:

[Variables]

x = col(1)
DepVar0 = col(3)

[Parameters]

k = 1 ' {{previous: −21.1382}}
a = 1 ' {{previous: −35.0724}}

[Equation]

f = a − ((k) × (x × ln(x))/(1 − x))
fit f to DepVar0

[Constraints]

[Options]

tolerance = 1e-010
stepsize = 1
iterations = 200

Number of Iterations Performed = 8

Fit of d13C Data of TCE in TCE Experiment

f = a + ((k) × ln(x))

**R**	**Rsqr**	**Adj Rsqr**	**Standard Error of Estimate**
0.993	0.986	0.985	0.970

	**Coefficient**	**Std. Error**	**t**	***p***
k	−12.208	0.456	−26.768	<0.0001
a	−26.742	0.424	−63.043	<0.0001

Confidence Intervals:

	**Coefficient**	**95% Conf-L**	**95% Conf-U**
k	−12.208	−13.225	−11.192
a	−26.742	−27.687	−25.797

Analysis of Variance:

	DF	SS	MS
Regression	2	4654.374	2327.187
Residual	10	9.407	0.941
Total	12	4663.781	388.648

Corrected for the mean of the observations:

	DF	SS	MS	F	*p*
Regression	1	674.050	674.050	716.522	<0.0001
Residual	10	9.407	0.941		
Total	11	683.457	62.132		

Statistical Tests:

Normality Test (Shapiro-Wilk) Passed (p = 0.1693)

W Statistic = 0.9022 Significance Level = 0.0500

Constant Variance Test Failed (p = 0.0186)

95% Confidence:

Row	Predicted	95% Conf-L	95% Conf-U
1	−26.742	−27.687	−25.797
2	−22.433	−23.149	−21.717
3	−21.244	−21.917	−20.571
4	−18.926	−19.553	−18.300
5	−15.054	−15.731	−14.377
7	−26.742	−27.687	−25.797
8	−19.850	−20.489	−19.212
9	−17.424	−18.051	−16.797
10	−14.747	−15.435	−14.060
11	−7.244	−8.350	−6.138
13	−26.742	−27.687	−25.797
14	−1.400	−2.932	0.132

Fit Equation Description:

[Variables]

x = col(1)
DepVar0 = col(2)

[Parameters]

k = 1 ' {{previous: −12.2085}}
a = 1 ' {{previous: −26.7423}}

[Equation]

f = a + ((k) × ln(x))
fit f to DepVar0

[Constraints]

[Options]

tolerance = 1e-010
stepsize = 1
iterations = 200

Number of Iterations Performed = 8

Fit of d13C Data of cis-DCE in TCE Experiment

f = a − ((k) × (x × ln(x))/(1 − x))

**R**	**Rsqr**	**Adj Rsqr**	**Standard Error of Estimate**
0.994	0.989	0.988	0.333

	**Coefficient**	**Std. Error**	**t**	***p***
k	−10.018	0.337	−29.689	<0.0001
a	−25.570	0.190	−134.575	<0.0001

Confidence Intervals:

	**Coefficient**	**95% Conf-L**	**95% Conf-U**
k	−10.018	−10.769	−9.266
a	−25.570	−25.993	−25.146

Analysis of Variance:

	**DF**	**SS**	**MS**
Regression	2	11211.965	5605.982
Residual	10	1.112	0.111
Total	12	11213.077	934.423

Corrected for the mean of the observations:

	DF	SS	MS	F	*p*
Regression	1	98.016	98.016	881.418	<0.0001
Residual	10	1.112	0.111		
Total	11	99.128	9.012		

Statistical Tests:

Normality Test (Shapiro-Wilk) Passed ($p = 0.6535$)

W Statistic = 0.9511 Significance Level = 0.0500

Constant Variance Test Passed ($p = 0.4981$)

95% Confidence:

Row	Predicted	95% Conf-L	95% Conf-U
2	−33.923	−34.262	−33.584
3	−33.500	−33.815	−33.186
4	−32.720	−32.995	−32.446
5	−31.545	−31.776	−31.315
6	−25.842	−26.248	−25.436
8	−33.024	−33.314	−32.735
9	−32.246	−32.500	−31.992
10	−31.459	−31.687	−31.231
11	−29.632	−29.854	−29.409
12	−25.776	−26.186	−25.366
14	−28.553	−28.809	−28.296
15	−26.975	−27.311	−26.638

Fit Equation Description:

[Variables]

x = col(1)
DepVar0 = col(3)

[Parameters]

k = 1 ' {{previous: −10.0176}}
a = 1 ' {{previous: −25.5696}}

[Equation]

f = a − ((k) × (x × ln(x))/(1 − x))
fit f to DepVar0

[Constraints]

[Options]

tolerance = 1e-010
stepsize = 1
iterations = 200

Number of Iterations Performed = 8

A7.2. Regressions of Figure 3

Fit of d37Cl PCE Data in PCE Experiment

$f = a + ((k) \times \ln(x))$

**R**	**Rsqr**	**Adj Rsqr**	**Standard Error of Estimate**
0.996	0.992	0.991	0.346

	**Coefficient**	**Std. Error**	**t**	***p***
k	−5.039	0.123	−40.855	<0.0001
a	−2.788	0.116	−23.977	<0.0001

Confidence Intervals:

	**Coefficient**	**95% Conf-L**	**95% Conf-U**
k	−5.039	−5.303	−4.774
a	−2.788	−3.037	−2.539

Analysis of Variance:

	**DF**	**SS**	**MS**
Regression	2	202.674	101.337
Residual	14	1.680	0.120
Total	16	204.354	12.772

Corrected for the mean of the observations:

	**DF**	**SS**	**MS**	**F**	***p***
Regression	1	200.344	200.344	1669.133	<0.0001
Residual	14	1.680	0.120		
Total	15	202.024	13.468		

Statistical Tests:

Normality Test (Shapiro-Wilk) Passed (p = 0.9985)

W Statistic = 0.9889 Significance Level = 0.0500

Constant Variance Test Passed (p = 0.8824)

95% Confidence:

**Row**	**Predicted**	**95% Conf-L**	**95% Conf-U**
1	−2.788	−3.037	−2.539
2	−2.382	−2.617	−2.146
3	−1.893	−2.114	−1.673
4	−1.110	−1.312	−0.908
5	−0.879	−1.076	−0.682
6	3.102	2.868	3.336
7	6.892	6.503	7.281
9	−2.788	−3.037	−2.539
10	−1.442	−1.651	−1.233
11	−1.020	−1.220	−0.820
12	0.128	−0.058	0.314
13	0.810	0.622	0.997
16	−2.788	−3.037	−2.539
17	−0.297	−0.486	−0.107
18	2.526	2.309	2.743
19	10.035	9.496	10.575

Fit Equation Description:

[Variables]

x = col(1)
DepVar0 = col(2)

[Parameters]

k = 1 ' {{previous: −5.03855}}
a = 1 ' {{previous: −2.78801}}

[Equation]

f = a + ((k) × ln(x))
fit f to DepVar0

[Constraints]

[Options]

tolerance = 1e-010
stepsize = 1
iterations = 200

Number of Iterations Performed = 7

Fit of d37Cl TCE Data in PCE Experiment

$f = a - ((k) \times (x \times \ln(x))/(1 - x))$

**R**	**Rsqr**	**Adj Rsqr**	**Standard Error of Estimate**
0.983	0.967	0.964	0.350

	**Coefficient**	**Std. Error**	**t**	***p***
k	−5.362	0.258	−20.806	<0.0001
a	1.811	0.165	10.962	<0.0001

Confidence Intervals:

	**Coefficient**	**95% Conf-L**	**95% Conf-U**
k	−5.362	−5.911	−4.813
a	1.811	1.459	2.163

Analysis of Variance:

	**DF**	**SS**	**MS**
Regression	2	74.968	37.484
Residual	15	1.834	0.122
Total	17	76.801	4.518

Corrected for the mean of the observations:

	**DF**	**SS**	**MS**	**F**	**p**
Regression	1	52.916	52.916	432.885	<0.0001
Residual	15	1.834	0.122		
Total	16	54.750	3.422		

Statistical Tests:

Normality Test (Shapiro-Wilk) Passed (p = 0.2820)

W Statistic = 0.9368 Significance Level = 0.0500

Constant Variance Test Passed (p = 0.1998)

95% Confidence:

Row	Predicted	95% Conf-L	95% Conf-U
2	−3.338	−3.627	−3.049
3	−3.089	−3.359	−2.820
4	−2.708	−2.950	−2.466
5	−2.599	−2.834	−2.365
6	−1.014	−1.195	−0.833
7	0.044	−0.174	0.261
8	1.489	1.165	1.813
10	−2.867	−3.120	−2.614
11	−2.665	−2.904	−2.426
12	−2.148	−2.357	−1.940
13	−1.863	−2.058	−1.667
14	1.245	0.941	1.549
15	1.568	1.237	1.899
17	−2.334	−2.553	−2.116
18	−1.212	−1.392	−1.031
19	0.649	0.392	0.906
20	1.480	1.157	1.804

Fit Equation Description:

[Variables]

x = col(1)
DepVar0 = col(4)

[Parameters]

k = 1 ' {{previous: −5.36208}}
a = 1 ' {{previous: 1.81095}}

[Equation]

f = a − ((k) × (x × ln(x))/(1 − x))
fit f to DepVar0

[Constraints]

[Options]

tolerance = 1e-010
stepsize = 1
iterations = 200

Number of Iterations Performed = 8

Fit of d37Cl Chloride Data in PCE Experiment

$f = a - ((k) \times (x \times \ln(x))/(1 - x))$

**R**	**Rsqr**	**Adj Rsqr**	**Standard Error of Estimate**
0.541	0.293	0.057	1.451

	**Coefficient**	**Std. Error**	**t**	***p***
k	−4.106	3.687	−1.114	0.3466
a	−14.512	1.439	−10.083	0.0021

Confidence Intervals:

	**Coefficient**	**95% Conf-L**	**95% Conf-U**
k	−4.106	−15.839	7.626
a	−14.512	−19.092	−9.931

Analysis of Variance:

	**DF**	**SS**	**MS**
Regression	2	1273.465	636.733
Residual	3	6.315	2.105
Total	5	1279.781	255.956

Corrected for the mean of the observations:

	**DF**	**SS**	**MS**	**F**	***p***
Regression	1	2.612	2.612	1.241	0.3466
Residual	3	6.315	2.105		
Total	4	8.927	2.232		

Statistical Tests:

Normality Test (Shapiro-Wilk) Passed (p = 0.9202)

W Statistic = 0.9774 Significance Level = 0.0500

Constant Variance Test Passed (p = 0.0500)

95% Confidence:

**Row**	**Predicted**	**95% Conf-L**	**95% Conf-U**
6	−16.675	−19.615	−13.735
7	−15.865	−17.942	−13.788
14	−14.945	−18.465	−11.425

18	−16.826	−20.088	−13.565
19	−15.402	−17.981	−12.822

Fit Equation Description:

[Variables]

x = col(1)
DepVar0 = col(6)

[Parameters]

k = 1 ' {{previous: −4.10628}}
a = 1 ' {{previous: −14.5118}}

[Equation]

f = a − ((k) × (x × ln(x))/(1 − x))
fit f to DepVar0

[Constraints]

[Options]

tolerance = 1e-010
stepsize = 1
iterations = 200

Number of Iterations Performed = 8

A7.3. Regressions of Figure 6

Fit of d37Cl TCE Data of TCE Experiment

f = a + ((k) × ln(x))

**R**	**Rsqr**	**Adj Rsqr**	**Standard Error of Estimate**
0.996	0.991	0.990	0.229

	**Coefficient**	**Std. Error**	**t**	***p***
k	−3.644	0.108	−33.864	<0.0001
a	1.244	0.100	12.427	<0.0001

Confidence Intervals:

	**Coefficient**	**95% Conf-L**	**95% Conf-U**
k	−3.644	−3.883	−3.404
a	1.244	1.021	1.467

Analysis of Variance:

	DF	SS	MS
Regression	2	232.350	116.175
Residual	10	0.524	0.052
Total	12	232.873	19.406

Corrected for the mean of the observations:

	DF	SS	MS	F	*p*
Regression	1	60.040	60.040	1146.768	<0.0001
Residual	10	0.524	0.052		
Total	11	60.563	5.506		

Statistical Tests:

Normality Test (Shapiro-Wilk) Passed (p = 0.8302)

W Statistic = 0.9633 Significance Level = 0.0500

Constant Variance Test Passed (p = 0.0795)

95% Confidence:

Row	Predicted	95% Conf-L	95% Conf-U
1	1.244	1.021	1.467
2	2.530	2.361	2.699
3	2.884	2.726	3.043
4	3.576	3.428	3.724
5	4.732	4.572	4.892
7	1.244	1.021	1.467
8	3.301	3.150	3.451
9	4.025	3.877	4.173
10	4.824	4.661	4.986
11	7.063	6.802	7.324
13	1.244	1.021	1.467
14	8.807	8.446	9.169

Fit Equation Description:

[Variables]

x = col(1)
DepVar0 = col(2)

[Parameters]

k = 1 ' {{previous: −3.64364}}
a = 1 ' {{previous: 1.24361}}

[Equation]

f = a + ((k) × ln(x))
fit f to DepVar0

[Constraints]

[Options]

tolerance = 1e-010
stepsize = 1
iterations = 200

Number of Iterations Performed = 8

Fit of d37Cl *cis*-DCE Data of TCE Experiment

f = a − ((k) × (x × ln(x))/(1 − x))

**R**	**Rsqr**	**Adj Rsqr**	**Standard Error of Estimate**
0.988	0.977	0.975	0.116

	**Coefficient**	**Std. Error**	**t**	***p***
k	−2.429	0.118	−20.610	<0.0001
a	2.939	0.066	44.289	<0.0001

Confidence Intervals:

	**Coefficient**	**95% Conf-L**	**95% Conf-U**
k	−2.429	−2.691	−2.166
a	2.939	2.791	3.087

Analysis of Variance:

	**DF**	**SS**	**MS**
Regression	2	42.921	21.461
Residual	10	0.136	0.014
Total	12	43.057	3.588

Corrected for the mean of the observations:

	DF	SS	MS	F	*p*
Regression	1	5.761	5.761	424.792	<0.0001
Residual	10	0.136	0.014		
Total	11	5.897	0.536		

Statistical Tests:

Normality Test (Shapiro-Wilk) Passed (p = 0.1070)

W Statistic = 0.8867 Significance Level = 0.0500

Constant Variance Test Passed (p = 0.1889)

95% Confidence:

Row	Predicted	95% Conf-L	95% Conf-U
2	0.914	0.795	1.032
3	1.016	0.906	1.126
4	1.205	1.109	1.301
5	1.490	1.410	1.570
6	2.873	2.731	3.014
8	1.131	1.030	1.233
9	1.320	1.232	1.409
10	1.511	1.431	1.591
11	1.954	1.876	2.032
12	2.889	2.746	3.032
14	2.216	2.126	2.305
15	2.598	2.481	2.716

Fit Equation Description:

[Variables]

x = col(1)
DepVar0 = col(4)

[Parameters]

k = 1 ' {{previous: −2.4287}}
a = 1 ' {{previous: 2.93881}}

[Equation]

f = a − ((k) × (x × ln(x))/(1 − x))
fit f to DepVar0

[Constraints]

[Options]

tolerance = 1e-010
stepsize = 1
iterations = 200

Number of Iterations Performed = 8

Fit of d37Cl Chloride Data of TCE Experiment

$f = a - ((k) \times (x \times \ln(x))/(1 - x))$

**R**	**Rsqr**	**Adj Rsqr**	**Standard Error of Estimate**
0.920	0.847	0.825	0.522

	**Coefficient**	**Std. Error**	**t**	***p***
k	−6.457	1.038	−6.223	0.0004
a	−1.216	0.673	−1.808	0.1136

Confidence Intervals:

	**Coefficient**	**95% Conf-L**	**95% Conf-U**
k	−6.457	−8.910	−4.003
a	−1.216	−2.807	0.375

Analysis of Variance:

	**DF**	**SS**	**MS**
Regression	2	259.643	129.821
Residual	7	1.904	0.272
Total	9	261.546	29.061

Corrected for the mean of the observations:

	**DF**	**SS**	**MS**	**F**	***p***
Regression	1	10.533	10.533	38.727	0.0004
Residual	7	1.904	0.272		
Total	8	12.437	1.555		

Statistical Tests:

Normality Test (Shapiro-Wilk) Passed ($p = 0.5600$)

W Statistic = 0.9379 Significance Level = 0.0500

Constant Variance Test Passed (p = 0.2428)

95% Confidence:

**Row**	**Predicted**	**95% Conf-L**	**95% Conf-U**
2	−6.601	−7.255	−5.946
3	−6.328	−6.906	−5.751
4	−5.825	−6.289	−5.362
5	−5.068	−5.486	−4.651
8	−6.021	−6.524	−5.519
9	−5.520	−5.942	−5.097
10	−5.013	−5.434	−4.591
11	−3.835	−4.515	−3.154
14	−3.139	−4.044	−2.234

Fit Equation Description:

[Variables]

x = col(1)
DepVar0 = col(6)

[Parameters]

k = 1 ' {{previous: −6.45696}}
a = 1 ' {{previous: −1.21636}}

[Equation]

f = a − ((k) × (x × ln(x))/(1 − x))
fit f to DepVar0

[Constraints]

[Options]

tolerance = 1e-010
stepsize = 1
iterations = 200

Number of Iterations Performed = 9

© 2014 by the authors; licensee MDPI, Basel, Switzerland. This article is an open access article distributed under the terms and conditions of the Creative Commons Attribution license (http://creativecommons.org/licenses/by/3.0/).
